# Entorhinal cortex vulnerability to human APP expression promotes hyperexcitability and tau pathology

**DOI:** 10.21203/rs.3.rs-3370607/v1

**Published:** 2023-11-06

**Authors:** Annie M Goettemoeller, Emmie Banks, Katharine E McCann, Prateek Kumar, Kelly South, Viktor J Olah, Christina C Ramelow, Duc M Duong, Nicholas T Seyfried, Srikant Rangaraju, David Weinshenker, Matthew JM Rowan

**Affiliations:** 1 Department of Cell Biology, Emory University School of Medicine, Atlanta, GA, 30322; 2 Department of Human Genetics, Emory University School of Medicine; 3 Department of Neurology, Emory University School of Medicine; 4 Department of Biochemistry, Emory University; 5 Center for Neurodegenerative Disease, Emory University School of Medicine; 6 GDBBS Graduate Program, Laney Graduate School, Emory University

**Keywords:** APP, inhibition, PV interneuron, hyperexcitability, lateral entorhinal cortex, Alzheimer’s Disease

## Abstract

Preventative treatment for Alzheimer’s Disease is of dire importance, and yet, cellular mechanisms underlying early regional vulnerability in Alzheimer’s Disease remain unknown. In human patients with Alzheimer’s Disease, one of the earliest observed pathophysiological correlates to cognitive decline is hyperexcitability^1^. In mouse models, early hyperexcitability has been shown in the entorhinal cortex, the first cortical region impacted by Alzheimer’s Disease^2–4^. The origin of hyperexcitability in early-stage disease and why it preferentially emerges in specific regions is unclear. Using cortical-region and cell-type- specific proteomics and patch-clamp electrophysiology, we uncovered differential susceptibility to human-specific amyloid precursor protein (hAPP) in a model of sporadic Alzheimer’s. Unexpectedly, our findings reveal that early entorhinal hyperexcitability may result from intrinsic vulnerability of parvalbumin interneurons, rather than the suspected layer II excitatory neurons. This vulnerability of entorhinal PV interneurons is specific to hAPP, as it could not be recapitulated with increased murine APP expression. Furthermore, the Somatosensory Cortex showed no such vulnerability to adult-onset hAPP expression, likely resulting from PV-interneuron variability between the two regions based on physiological and proteomic evaluations. Interestingly, entorhinal hAPP-induced hyperexcitability was quelled by co-expression of human Tau at the expense of increased pathological tau species. This study suggests early disease interventions targeting non-excitatory cell types may protect regions with early vulnerability to pathological symptoms of Alzheimer’s Disease and downstream cognitive decline.

Alzheimer’s Disease (AD) is the most prevalent neurodegenerative disease, yet current treatments are unable to prevent its initiation and progression. Although brain regions of early vulnerability have been known for over 30 years^5^, our understanding of what makes certain areas more susceptible remains unknown. The first cortical region to display pathology and degeneration in AD is the Lateral Entorhinal Cortex (LEC)^2,5–7^. Notably, landmark studies identified Layer II (LII) neurons cells as highly vulnerable to early neurodegeneration with up to 60% cell death in mild AD patients and up to 90% in severe cases^2^. More recently, LII LEC principal neurons were also characterized as a cell population exhibiting amyloid pathology^7^. However, the distinctive features which impart vulnerability to neurons in the LEC AD remain unclear. Uncovering region-specific cellular mechanisms could improve our understanding of the initiating factors in the AD cascade and are imperative in determining potential interventions at a time when subsequent cognitive decline and neurodegeneration might still be prevented.

Hyperexcitability is one of the earliest pathophysiological biomarkers in the human AD brain, and its emergence correlates with severity of cognitive decline in individuals^1^. Hyperexcitability is also observed in recordings from *in vivo* and *in vitro* models of AD pathology^8–14^, arising prior to amyloid plaque deposition^15^ and likely contributing to spine degeneration^16^. Interestingly, hypermetabolism^3^ and hyperexcitability^4,11^ emerged in the LEC of a sporadic AD mouse model before spreading to other regions^6^. It is unclear whether cell-intrinsic changes in principal neuron excitability or other forms of circuit dysfunction are responsible for aberrant LEC activity in early AD. Hyperexcitability may also arise due to changes in local circuit inhibition from GABAergic interneurons, with several lines of evidence demonstrating impaired inhibitory tone^3,8,11^, most notably from fast-spiking parvalbumin+ (PV) interneurons^9,12,15^. Whether the basal properties of PV interneurons in the LEC confer functional vulnerability with respect to PV cells in other regions is unknown. Thus, observing baseline cellular and regional differences coupled with adult-onset, region-specific APP or Tau expression is imperative to properly dissect inherent vulnerabilities underlying susceptibility of the LEC to early AD pathology.

## PV interneurons in an AD-vulnerable region are functionally and molecularly distinct

**Figure 1.**
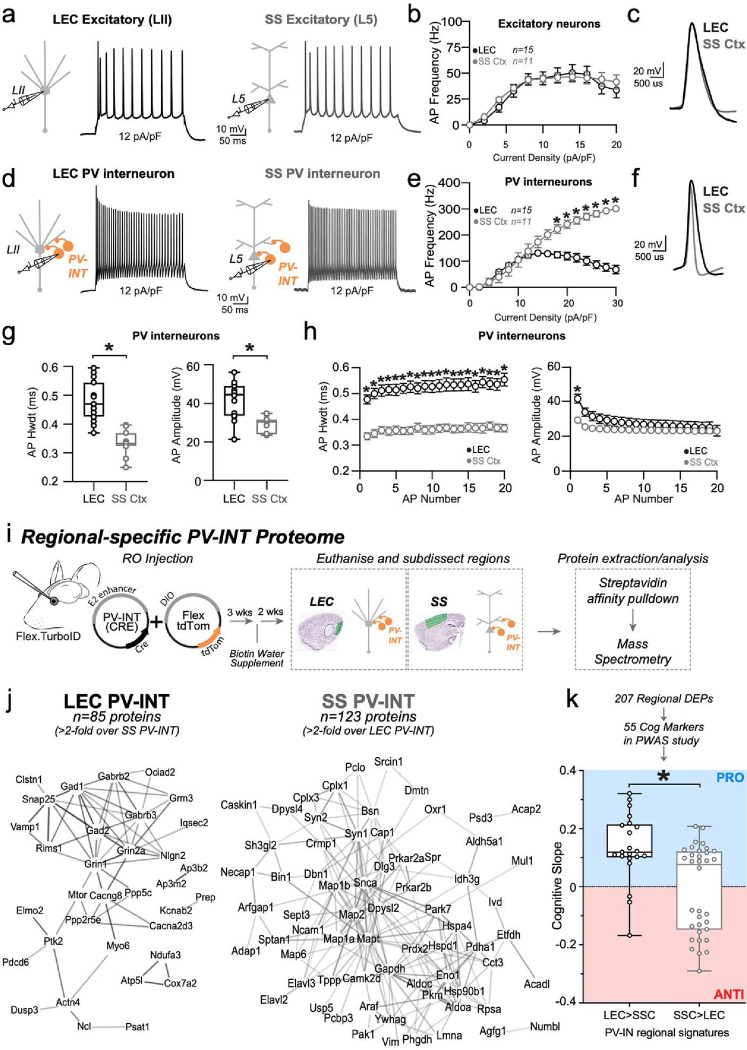
PV-INTs in an AD vulnerable region display reduced baseline firing **a,d.** Graphical summary of AAV.E2.tdTom stereotactic injection in either the Lateral Entorhinal Cortex or Somatosensory cortex. PV-interneurons were fluorescently targeted for whole-cell current clamp recordings (d) as well as nearby excitatory cells (a). AP firing elicited firing by square pulse current injections of varying magnitude normalized to cellular capacitance during recording in excitatory cells (a) and PV-interneurons (d) mice from L2 LEC (left) and L5 SS Ctx (Right) at 12 pA/pF. **b.** Group data summary of AP firing frequency in WT mice. Excitatory cells between LEC and SS Ctx showed no difference in AP Frequency (Hz) Ctx (LEC: Max: 50.42±5.638 Hz, SS Ctx: Max: 46.35±5.514 Hz, p=0.4645). **c.** AP waveforms of excitatory cells were compared at 12 pA/pF square pulse injections in WT mice from L2 LEC and L5 SS Ctx. Aps from the 1^st^ spike in the train are superimposed for comparison. **e.** Group data summary of AP firing frequency in WT mice. PV interneurons in L2 LEC show a strong reduction in AP max firing frequency at higher current densities when compared to PV interneurons of L5 SS Ctx (LEC: Max: 131.6±11.48 Hz, SS Ctx: Max: 301.1±27.59 Hz, p=<0.0001 for 16 pA/pF and above). **f.** AP waveforms of tdTom+ PV interneurons were compared at 12 pA/pF square pulse injections in WT mice from L2 LEC and L5 SS Ctx. Aps from the 1^st^ spike in the train are superimposed for comparison. **g.** Summary data of AP properties. L2 LEC PV interneurons display a significantly increased AP amplitude (LEC: 41.86±2.66 pA, SS: 28.75±1.30 pA, p=0.0002, t= 4.825, df=24, two-tailed unpaired t-test) and AP Hwdt (LEC: 0.4780 ± 0.01767 ms, SS: 0.3342 ± 0.01314 ms, p=<0.0001, t=6.103, df=25) for the first AP of the spike train. Individual data points and box plots are displayed. Significance is defined as p<0.05, unpaired t-tests. **h.** Relationship between AP amplitude or width, in WT mice and AP # during spike trains elicited with a 12 pA/pF current injection. **i.** Experimental approach for Regional-specific PV-interneuron Proteomes: E2 enhancer Cre AAV was retro-orbitally delivered to WT (Control) or Rosa26TurboID/wt (PV-CIBOP) mice (n=3 per genotype, including males and females) followed by 3 weeks of Cre-mediated recombination, and 2 additional weeks of biotin supplementation (drinking water). The brain was then microdissected into LEC and SS Ctx and prepared for biochemical studies. **j.** STRING analysis of PV-enriched proteins for LEC PV-INTs (left) and SS Ctx PV-INTs (right) (>2-fold enriched over other region) shows synaptic receptors, synaptic vesicle and exocytosis related proteins including GAD1/2, GABAb2/3, and complexins. **k.** Enrichment of PWAS-identified proteins associated with cognitive slope in LEC (left) or SS Ctx (right) PV-enriched proteomic signatures. Cognitive slope was estimated in ROSMAP cases. Positive slope indicates cognitive stability or resilience when proteins are present while a negative slope indicates cognitive decline when proteins are present. Proteins positively correlated with cognitive slope are referred to as pro-resilience proteins while those negative correlated with cognitive slope are anti-resilience proteins. Enrichment of pro-resilience and anti-resilience proteins in PV-enriched proteins identified by CIBOP was assessed after weighting based on strength of association between proteins and cognitive slope. (LEC: 0.1332± 0.02578 and SS Ctx: −0.01083±0.02596; p=0.0011, t=3.713, df=53, two-tailed Mann Whitney test). For b, e, and h: For all summary graphs, data are expressed as mean (± SEM). Statistical significance is denoted as *=p<0.05, as determined by Two-way ANOVA with Sidak’s multiple comparison test. For all summary graphs, data are expressed as mean (± SEM). Also see Extended Data Figure 1 for related analyses and datasets.

We first compared active and passive features of excitatory neurons in AD-vulnerable and non-vulnerable cortical regions. Excitatory neurons in LII of Lateral Entorhinal Cortex (LEC) (highly vulnerable to early AD pathology^7^) and L5 pyramidal cells (PCs) in Somatosensory Cortex (SS Ctx) of wild type (WT) mice were chosen for comparison, as each represent projection output neurons and are innervated by similar dominant inhibitory networks^17^. Despite differences in their dendritic anatomy, axonal projections, and overall local circuit operations, these two cell types showed striking overlap in their firing capacity, AP waveforms, and most other biophysical features (Fig 1a–c), with only slight biophysical differences noted (Extended Data Fig. 2). Because different cortical regions perform operations over non-overlapping frequency domains, we hypothesized that differences in the intrinsic excitability of inhibitory interneurons might help tune circuit activity locally. Thus, we assessed physiological phenotypes of ‘fast-spiking’ PV interneurons in each region, using an unbiased, PV-specific enhancer-AAV fluorescent targeting approach^18^. Surprisingly, PV interneurons in the LEC maximally fired at only half the rate of SS Ctx PV interneurons (Fig. 1d,e), likely due to their far broader action potentials with respect to PV interneurons recorded from SS Ctx (Fig. 1f–h)^17^. The first action potential of each AP train was also larger in amplitude in the LEC PV interneurons (Fig. 1f–h). Furthermore, resting membrane potential and AP threshold were significantly different for PV cells when compared by region (Extended Data Fig. 2, bottom). Despite expressing similar passive features in LEC and SS Ctx (e.g., membrane capacitance; 70.17 ± 5.46 pF vs. 71.91 ± 9.51 pF; LEC vs SS respectively), their starkly divergent excitability suggests unique molecular signatures which may also underlie differential vulnerability in AD and other diseases.

**Extended Data Figure 1.**
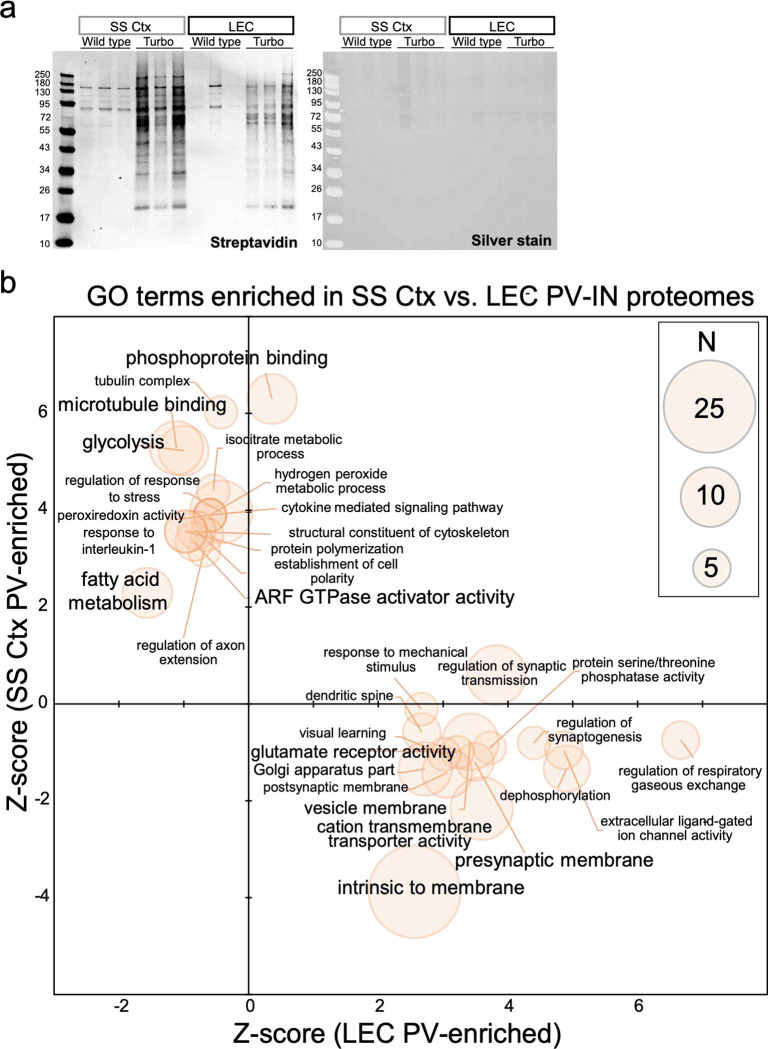
Enriched biotinylated PV-interneuron proteins from the SS Ctx and LEC are neuron-specific **a.** Western blot (left) and silver stain (right) visualization of enriched biotinylated proteins in PV-interneurons (IN) from the SS Ctx and LEC after streptavidin-pulldown and elution of biotinylated proteins from a 10% aliquot of beads. **b.** GSEA of ^3^ 2-fold enriched biotinylated PV-interneuron proteins from the SS Ctx and LEC from PV-CIBOP mice, as compared to a reference protein list of both regions (n = 807) showed enrichment of synaptic and neuronal proteins confirming neuronal labeling. The orange dot size represents the number of gene symbols represented in each GO term. WT (Control) or or Rosa26TurboID/wt (PV-CIBOP) mice (n=3 per genotype, including males and females).

We next sought to examine differences in the molecular signature of PV interneurons in each of these regions. Single-neuron transcriptomics is a sound method for uncovering molecular diversity between different brain cell types. Nonetheless, the functional relevance of these studies is limited by substantial discordance between mRNA and protein in neurons^19^. Thus, we opted to isolate the native-state proteomes of PV interneurons from each region using our recently developed neuron-type-specific TurboID method^20^ (Fig. 1i). This was achieved through systemic AAV injections to achieve whole-cortex expression of a PV-specific, Cre-expressing enhancer-AAV in Flex.TurboID mice^21^ followed by region-specific microdissection (Fig. 1i; Extended Data Fig. 1a). Over 800 proteins were biotinylated in PV interneurons in each region, of which nearly two hundred proteins showed region-specific differential abundances (unadjusted p<0.05 n=207; n=185 below the permFDR 0.05 threshold; Extended Data Datasheet 1; Fig. 1j). Generally, LEC PV interneuron proteomes showed biased enrichment in transmembrane and synaptic ion channels and transporters, while SS PV interneuron proteomes showed biased enrichment in microtubule binding, glycolysis, and fatty acid metabolism-related proteins (Extended Data Fig. 1b).

We next considered whether PV interneuron proteins differentially expressed by region (Fig. 1j) were representative of proteins associated with cognitive stability during aging. To achieve this, we used data from a protein-wide association study of cognitive resilience from human brain samples (Religious Orders Study and the Rush Memory and Aging Project; ‘ROSMAP’^22^) (Fig. 1k). Wild-type LEC PV interneurons displayed significantly more ‘pro-resilience’-associated proteins (p=0.0011; Mann-Whitney test; Fig. 1k), suggesting patients with preserved LEC PV interneuron integrity may exhibit increased resilience to cognitive decline. Thus, we next explored whether this molecular signature conferred resilience to PV interneurons in the LEC following an adult-onset induction of AD-related pathology.

## Adult-onset human APP expression reduces PV interneuron excitability specifically in LEC

**Figure 2.**
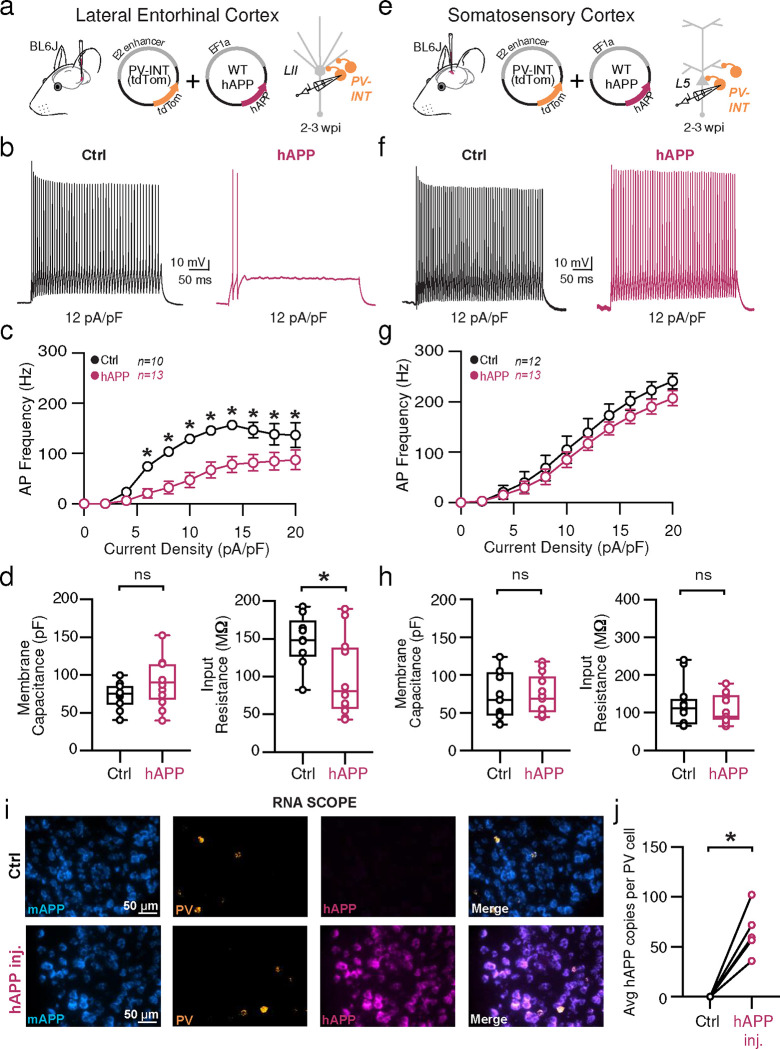
Adult-onset human APP expression reduces LEC PV interneuron excitability **a.** Graphical summary of AAV.E2.tdTom and AAV.EF1a.hAPP (or for Ctrl, saline) stereotactic injection in the Lateral Entorhinal Cortex. PV-interneurons were fluorescently targeted (tdTom+) for whole-cell current clamp recordings. **b.** AP firing elicited firing by square pulse current injections of varying magnitude normalized to cellular capacitance during recording in tdTom+ PV-INT from L2 LEC at 12 pA/pF. **c.** Group data summary of AP firing frequency in L2 LEC from Ctrl (black) and hAPP injected mice (magenta). LEC PV interneurons from hAPP injected mice show a significant reduction in AP Frequency (Hz) when compared to Ctrl(Ctrl: Max: 156.6 ±13.52 Hz, hAPP: Max: 91.84± 8.736 Hz). **d.** Summary data of AP properties. L2 LEC PV interneurons after hAPP injection display a significantly decreased input resistance (Ctrl: 145.7 ± 11.61 MΩ, hAPP: 88.78 ± 15.11 MΩ, p=0.0138, t=2.727, df=21) and an insignificant increase in membrane capacitance (Ctrl: 68.83 ± 5.336 pF, hAPP: 90.21 ± 9.771 pF, p=0.0708, t=1.921, df=21). **e.** Graphical summary of AAV.E2.tdTom and AAV.EF1a.hAPP (or for Ctrl, saline) stereotactic injection in the Somatosensory Cortex. PV-interneurons were fluorescently targeted (tdTom+) for whole-cell current clamp recordings. **f.** AP firing elicited firing by square pulse current injections of varying magnitude normalized to cellular capacitance during recording in tdTom+ PV-INT from L5 SS Ctx at 12 pA/pF. **g.** Group data summary of AP firing frequency in L5 SS Ctx from Ctrl (black) and hAPP injected mice (magenta). SS Ctx PV interneurons from hAPP injected mice show no significant change in AP Frequency (Hz) when compared to Ctrl (Ctrl: Max: 301.1 ±27.59 Hz, hAPP: Max: 257.2±24.06 Hz). **h.** Summary data of AP properties. SS Ctx interneurons after hAPP injection display an unchanged Membrane Capacitance (Ctrl: 71.91 ± 9.514, hAPP: 73.14 ± 7.327, p=0.9180, t=0.1041, df=23) and input resistance (Ctrl: 121.2 ± 17.14, hAPP: 109.1 ± 10.56, p=0.5475, t=0.6106, df=23). **i.** RNAscope representative images at 40x magnification for Ctrl injected (top) and hAPP injected mice (bottom): mAPP mRNA (cyan), Parvalbumin mRNA (gold), human APP mRNA (magenta), and a final merged image. **j.** RNAscope quantification for hAPP copies per PV+ cell comparing control to hAPP injected. hAPP injected show a significant increase in hAPP copies per PV+ cell (p=0.0039, t=5.987, df=4; two-tailed paired t-test). For all summary graphs, data are expressed as mean (± SEM). For c, g, and i: Statistical significance is denoted as *=p<0.05, as determined by Two-way ANOVA with Sidak’s multiple comparison test. For d, h: Individual data points and box plots are displayed. Statistical significance is denoted as *=p<0.05, as determined by two-tailed unpaired t-test.

**Figure 3.**
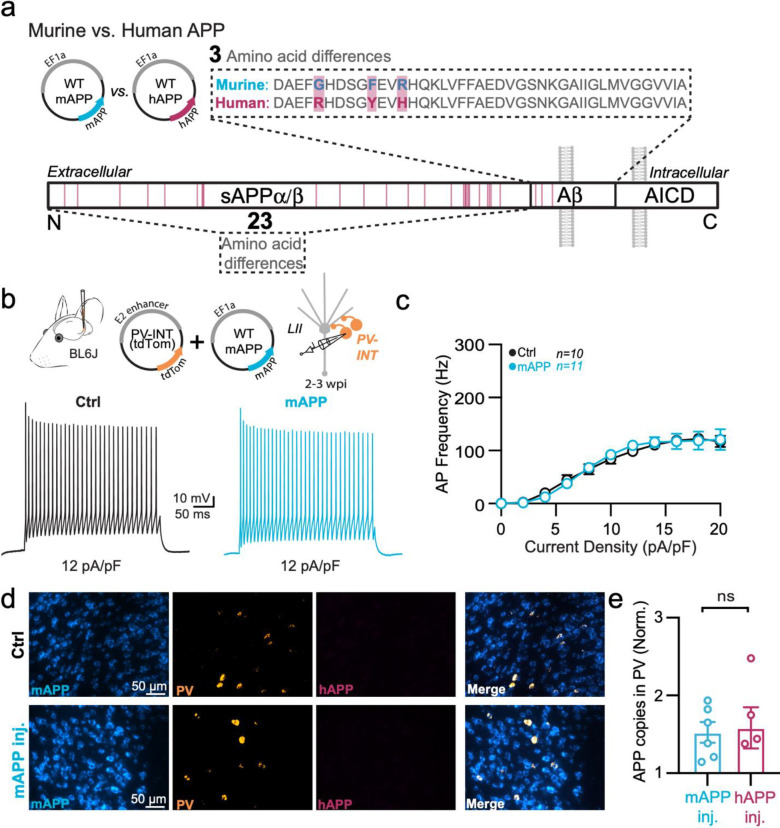
Murine APP does not affect PV interneuron physiology **a.** Pictorial representation of differing amino acids between murine APP and human APP proteins; 26 different amino acids in total, 3 of which are in the amyloid-beta segment of the protein. **b.** Graphical summary of AAV.E2.tdTom and AAV.EF1a.mAPP (or for Ctrl, saline) stereotactic injection in the Lateral Entorhinal Cortex. PV interneurons were fluorescently targeted for whole-cell current clamp recordings**.** AP firing elicited by square pulse current injections of varying magnitude normalized to cellular capacitance during recording in PV interneurons from Ctrl (left) and mAPP injected (right) L2 LEC at 12 pA/pF. **c.** Group data summary of AP firing frequency in Ctrl and mAPP injected mice. PV interneurons between Ctrl and mAPP injected showed no difference in AP Frequency (Hz) (Ctrl: Max: 122.3±11.11 Hz, mAPP: Max: 120.6 ± 11.50 Hz, p=0.9525). Statistical significance is denoted as *=p<0.05, as determined by Two-way ANOVA with Sidak’s multiple comparison test. **d.** RNAscope representative images at 40x magnification for Ctrl (top) and mAPP (bottom) injected mice: mAPP mRNA (cyan), Parvalbumin mRNA (gold), human APP mRNA (magenta), and a final merged image. **e.** RNAscope quantification for APP copies per PV+ cell with APP injected (mAPP or hAPP) each normalized to their contralateral hemisphere average endogenous murine APP copy per PV+ cell. mAPP injected and hAPP injected mice show similar increases in increased APP expression. copies per PV+ cell (p=0.8359, t=0.2133, df=9; two-tailed unpaired t-test).

Traditional rodent models of AD express various (typically mutant) forms of hAPP (and related processing proteins), with transgene expression beginning while neuronal circuits are still maturing, and also in a brain-wide fashion. To eliminate the substantial network effects of hAPP during development^23^ and to assess inherent vulnerability of individual areas independently, we used an adult-onset, region-specific AAV approach. To explore whether differences in basal excitability and proteomic signatures of PV interneurons described early conferred region-specific vulnerability in an AD pathology context, we virally expressed wild-type hAPP in either the LEC or SS Ctx in 8–12 week old (adult) mice. Full length hAPP (hAPP 770) (NM_000484.4), an isoform with increased expression in human AD^24,25^ was expressed using the pan-neuronal EF1a promoter (Figure 2a; AAV.Ef1a.hAPP). We assessed the impact of this hAPP isoform on PV interneurons in the LEC and SS Ctx independently after 2–3 weeks of expression. In LEC PV interneurons, we observed a dramatic reduction in PV interneuron firing (Fig. 2b,c) likely related to related to a reduction in input resistance (Fig. 2d), as no other relevant factors (e.g., AP waveform, RMP, AP threshold) (Extended Data Fig. 3c,d) were affected. By contrast, PV interneurons in the SS Ctx displayed no change in firing rate (Fig. 3g; Extended Data Fig. 3c–e) despite a slight increase in AP threshold (Extended Data Fig. 4). Using unsupervised clustering of LEC ‘fast-spiking’ interneuron biophysical features, control- and hAPP-expressing PV interneurons clustered separately (Extended Data Fig. 8b). The presence of hAPP mRNA and protein was confirmed in PV neurons 2–3 weeks after injection (Fig. 2i–j; Extended Data Fig. 4) with RNAscope and PV-specific flow cytometry. Together, the intrinsic excitability of PV interneurons was significantly reduced in the LEC, but not SS Ctx, following hAPP expression.

## Adult-onset murine APP expression does not affect PV interneuron physiology

Several studies of different mouse models of APP-related pathology report altered intrinsic excitability in GABAergic intereneurons^9–12,15^. Whether this is simply a result of hAPP overexpression^26^ during development or effects of its downstream cleavage products remain controversial. To address this, we next injected a virus containing full-length murine APP (mAPP) (NM_001198823.1) (Fig. 3a; AAV.Ef1a.mAPP) into the LEC. Despite a significant increase of mAPP expression over endogenous background levels (Fig. 3d), the robust changes in PV interneuron firing and input resistance seen following hAPP expression (Fig. 2) were lacking following 2–3 weeks of viral mAPP expression (Fig. 3b,c; Extended Data Fig. 4b–e). Importantly, RNAscope studies confirmed that the magnitude of AAV-induced mAPP expression was similar to that of hAPP in earlier experiments (Figure 3e), indicating that the differential physiological effects were not due to variability in hAPP or mAPP expression. Thus, hAPP-induced dysfunction of LEC PV interneurons cannot be explained by overexpression of APP alone.

## Adult-onset human APP expression does not affect excitatory cell intrinsic properties

**Figure 4.**
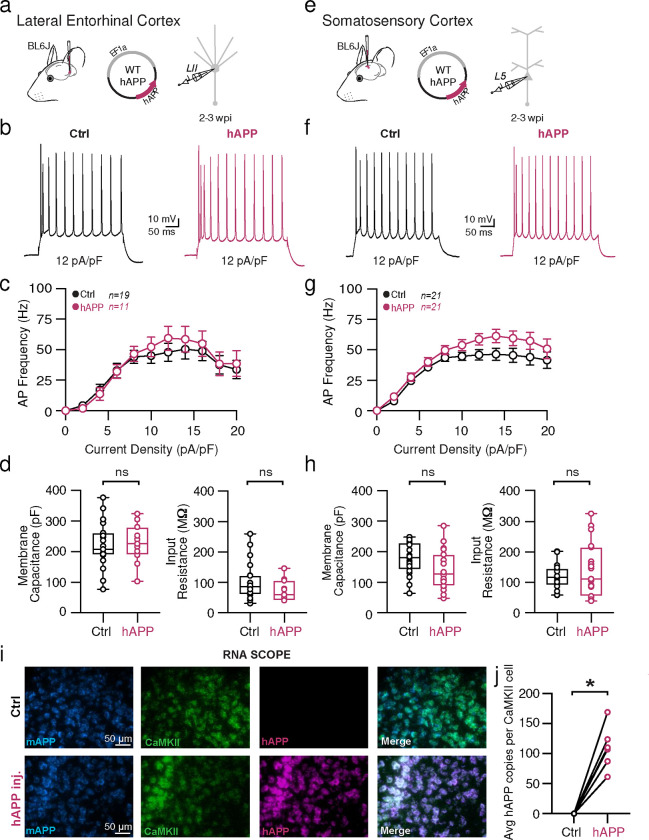
Adult-onset human APP expression does not alter excitatory neuron physiology **a.** Graphical summary of AAV.EF1a.hAPP (or for Ctrl, saline) stereotactic injection in the Lateral Entorhinal Cortex. Excitatory cells were targeted for whole-cell current clamp recordings. **b.** AP firing elicited firing by square pulse current injections of varying magnitude normalized to cellular capacitance during recording in Ctrl and hAPP injected L2 LEC excitatory cells from at 12 pA/pF. **c.** Group data summary of AP firing frequency in L2 LEC from Ctrl (black) and hAPP injected mice (magenta). Excitatory neurons in L2 LEC from hAPP injected mice show no alteration in AP Frequency (Hz) when compared to Ctrl (Ctrl: Max: 50.42 ± 5.63 Hz, hAPP: Max: 59.43 ± 6.56 Hz, p=0.9970, df=28). **d.** Summary data of AP properties. L2 LEC excitatory cells after hAPP injection display an unchanged Membrane Capacitance (p=0.8294, t=0.2715) as well as an unchanged input resistance (p=0.1452, t=1.498, df=28). **e.** Graphical summary of AAV.EF1a.hAPP (or for Ctrl, saline) stereotactic injection in the Somatosensory Cortex. Excitatory neurons in L5 were targeted for whole-cell current clamp recordings. **f.** AP firing elicited firing by square pulse current injections of varying magnitude normalized to cellular capacitance during recording in excitatory cells from L5 SS Ctx at 12 pA/pF. **g.** Group data summary of AP firing frequency in L5 SS Ctx from Ctrl (black) and hAPP injected mice (magenta). SS Ctx excitatory neurons from hAPP injected mice show no significant change in AP Frequency (Hz) when compared to Ctrl (Ctrl: Max: 46.35 ± 5.38 Hz, hAPP: Max: 61.43 ± 6.78 Hz, p>0.05, df=40). **h.** Summary data of AP properties. SS Ctx interneurons after hAPP injection display an unchanged Membrane Capacitance and input resistance (Ctrl: 176.9 ± 11.58, hAPP: 140.5 ± 14.31, p=0.0552, t=1.978, df=40, two-tailed unpaired t-test). **i.** RNAscope representative images at 40x magnification for Ctrl injected (top) and hAPP injected mice (bottom(: mAPP mRNA (cyan), CaMKIIa mRNA (green), human APP mRNA (magenta), and a final merged image. **j.** RNAscope quantification for hAPP copies per CaMKIIa+ cell comparing control to hAPP injected. hAPP injected show a significant increase in hAPP copies per CaMKIIa+ cell (p=0.0007, t=7.415, df=5; two-tailed paired t-test). For all summary graphs, data are expressed as mean (± SEM). For c, g: Statistical significance is denoted as *=p<0.05, as determined by Two-way ANOVA with Sidak’s multiple comparison test. For d, h: Individual data points and box plots are displayed. Statistical significance is denoted as *=p<0.05, as determined by two-tailed unpaired t-test.

Because recent studies using different mouse models of APP/Aβ pathology report altered intrinsic excitability of excitatory neurons^23,27^, we also assessed the effects of 2–3 weeks of hAPP expression on principal excitatory cells in the LEC and SS Ctx. Consistent with unaltered PV firing in SS Ctx, no change in firing frequency or passive properties were noted pyramidal cells in the SS Ctx (Fig. 4g–h). Surprisingly, we also observed no impact of hAPP on AP firing of LII LEC excitatory neurons (Fig. 4c). Further, membrane capacitance was unperturbed (Fig. 4d) suggesting no major alterations to LII cellular morphology. A modest, but significant increase in dV/dt max was noted in LEC LII principal cells (Extended Data Fig. 6c), potentially via an hAPP-dependent modulation of Na_v_ channels in these cells. Importantly, RNAscope experiments confirmed increased hAPP expression in CaMKIIa+ cells (Fig. 4i–j), indicating that our AAV also targeted excitatory neurons as expected. Using principal component analysis (PCA) of several excitatory cell biophysical features from LEC recordings, clusters could be separated based on input resistance, membrane time constant, and resting membrane potential. These clusters likely arise due to sampling of both LII fan cells and LII pyramidal cells^28^, suggesting our population of principal cells likely included both cell types (Extended Data Fig. 8a). When assessed, these excitatory populations showed no differential clustering following hAPP expression (Extended Data Fig. 8a). Together, these results indicate that principal neurons are more resistant to changes in their intrinsic excitability following adult-onset hAPP expression compared to PV interneurons.

## hAPP expression induces basal hyperexcitability in the LEC but not SS Ctx

**Figure 5.**
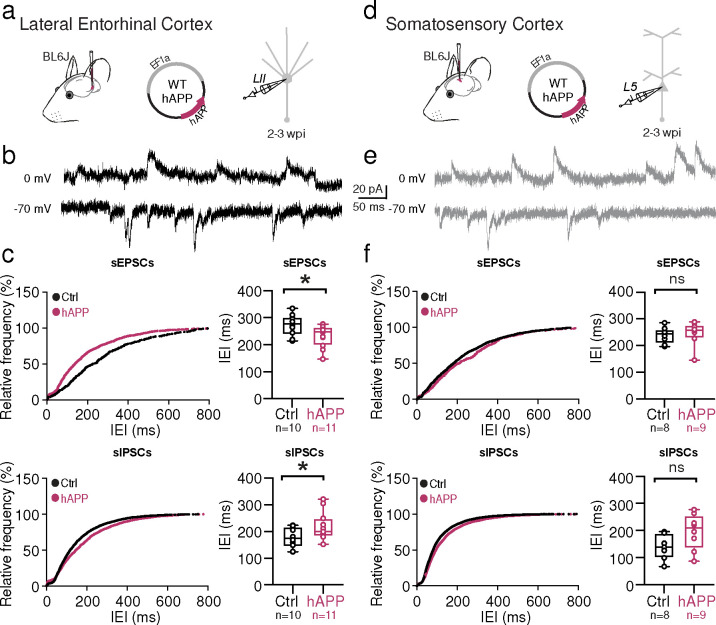
Human APP expression induces hyperexcitability in the LEC but not SS Ctx **a.** Graphical summary of AAV.EF1a.hAPP (or for Ctrl, saline) stereotactic injection in the Lateral Entorhinal Cortex. Excitatory cells were targeted for whole-cell voltage-clamp recordings. **b.** Spontaneous events obtained by holding cell voltage at 0 mV (inhibitory post-synaptic currents, IPSCs [top]) and −70 mV (excitatory post-synaptic currents, EPSCs [bottom]), interleaved. **c. Top:** Cumulative distribution curve for spontaneous EPSCs in the LEC showing the relationship of relative frequency of events to the inter-event interval (left). Quantified averages of event IEI are displayed for each cell as individual data points and compared between Ctrl (black) and hAPP injected (magenta) conditions (right). L2 LEC sEPSCs show a significant reduction in the IEI between events (231.7 ± 12.25 ms, 272.7 ± 12.24 ms, hAPP and Ctrl respectively, p=0.029, t=2.361, df=19, two-tailed unpaired t-test). See Extended Data Fig. 8 for mEPSC data. **Bottom:** Cumulative distribution curve for spontaneous IPSCs in the LEC showing the relationship of relative frequency of events to the inter-event interval (left). Quantified averages of event IEI are displayed for each cell as individual data points and compared between Ctrl (black) and hAPP injected (magenta) conditions (right). L2 LEC sIPSCs show a significant increase in the IEI between events (219.9 ± 15.84 ms, 177.3 ± 12.02 ms, hAPP and Ctrl respectively, p=0.047, t=2.097, df=19, two-tailed unpaired t-test). See Extended Data Fig. 8 for mIPSC data. **d.** Graphical summary of AAV.EF1a.hAPP (or for Ctrl, saline) stereotactic injection in the Somatosensory Cortex. Excitatory cells were targeted for whole-cell voltage-clamp recordings. **e.** Spontaneous events obtained by holding cell voltage at 0 mV (IPSCs [top]) and −70 mV (EPSCs [bottom]), interleaved. **f. Top:** Cumulative distribution curve for spontaneous EPSCs in the SS Ctx showing the relationship of relative frequency of events to the inter-event interval (left). Quantified averages of event IEI are displayed for each cell as individual data points and compared between Ctrl (black) and hAPP injected (magenta) conditions (right). L5 SS Ctx sEPSCs show no change in the IEI between events (p=0.7372, t=0.3450, df=15; two-tailed unpaired t-test). See Extended Data Fig. 8 for mEPSC data. **Bottom:** Cumulative distribution curve for spontaneous IPSCs in the SS Ctx showing the relationship of relative frequency of events to the inter-event interval (left). Quantified averages of event IEI are displayed for each cell as individual data points and compared between Ctrl (black) and hAPP injected (magenta) conditions (right). L5 SS Ctx sIPSCs show no change in the IEI between events (p=0.0812, t=1.890, df=15; two-tailed unpaired t-test). See Extended Data Fig. 8 for mIPSC data.

Although we observed no alterations in the intrinsic excitability of excitatory cells in either region following hAPP expression, we wanted to assess whether the changes in PV interneuron biophysics in LEC had an impact on local circuit activity. To examine this at population level, we continuously acquired spontaneous post-synaptic currents from principal cells in either region (Fig. 5). In the LEC, sIPSC frequency (Fig. 5a,b) was significantly decreased (increase in the mean inter-event interval [IEI]) after 2–3 weeks of hAPP expression (Fig. 5c). This was consistent with changes in intrinsic PV excitability observed earlier. In an apparent response to this reduced inhibitory tone, sEPSC frequency increased in the LEC following hAPP expression (Fig. 5c). In contrast to the LEC, recording from SS Ctx (Fig. 5d,e) revealed no change in sIPSC or sEPSC frequency following hAPP expression (Fig. 5f), in agreement with the lack of changes in intrinsic excitability in the SS Ctx shown earlier. Spontaneous and miniature (excitatory or inhibitory) synaptic amplitudes in the LEC and SS Ctx were unchanged in either region (Extended Data Fig. 9), indicating that postsynaptic receptor alterations did not arise in excitatory neurons following short-term adult-onset hAPP expression. mIPSC and mEPSC frequencies were also unaltered, suggesting no change in the number of inhibitory or excitatory synapses at this point (Extended Data Fig. 9b). Together, these results indicate that following adult-onset hAPP expression, basal circuit activity in the LEC, but not SS Ctx, becomes hyperexcitable, likely resulting from a region-specific PV interneuron vulnerability.

## hTau co-expression with hAPP quells LEC hyperexcitability at the cost of increased pathological tau species

**Figure 6.**
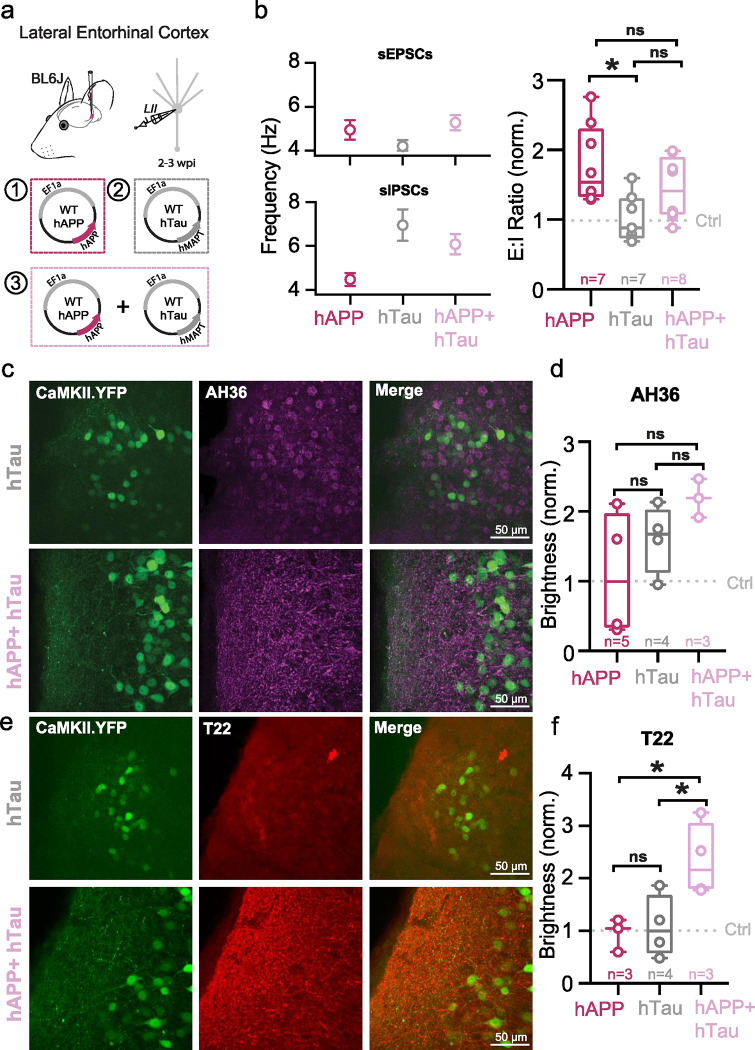
hTau co-expression with hAPP quells hyperexcitability but increases pathological tau **a.** Graphical summary of AAV.EF1a.hAPP, AAV.EF1a.MAPT (hTau), or co-injected AAV.EF1a.hAPP with AAV.EF1a.MAPT stereotactic injection in the Lateral Entorhinal Cortex. Excitatory cells were targeted for whole-cell voltage-clamp recordings. **b.** Spontaneous events obtained by holding cell voltage at −70 mV (excitatory post-synaptic currents, EPSCs [top]) and 0 mV (inhibitory post-synaptic currents, IPSCs [bottom]), interleaved. Quantified averages of event frequency are displayed for each cell normalized to Ctrl values as a ratio of EPSC Frequency to IPSC frequency and compared between hAPP injected (magenta), hTau injected (gray) and hAPP + hTau co-injected (pink) conditions. L2 LEC injected with hAPP showed a significantly elevated E:I ratio compared to hTau injected (p=0.0136, df=20). hAPP and hTau co-injected E:I ratio was not significantly different from hAPP injected (p=0.3323, df=20) or hTau injected (p=0.2175, df=20). For all summary graphs, data are expressed as mean (± SEM). Statistical significance is denoted as *=p<0.05, as determined by an Ordinary one-way ANOVA with Multiple comparisons. **c,e.** IHC representative images at 60x magnification for hTau (top) or hAPP+hTau (bottom) injected mice (for Ctrl or hAPP injected, see Extended Data Fig. 10) with staining for either AH36 (c) or T22 (e). **d.** hAPP, hTau, and hAPP+hTau were analyzed for AH36 brightness in the first 100 um of every slice. AH36 brightness was normalized to CaMKII.eYFP brightness to control for any potential variability in viral expression. All groups were then normalized to the Ctrl injected condition. hAPP+hTau showed the highest level of AH36 brightness, although it was not significant over hAPP (p=0.1267) or hTau (p=0.4900) (df=8, One-Way ANOVA with Multiple Comparisons). hAPP and hTau were also not significantly different (p=0.5328). **e.** hAPP, hTau, and hAPP+hTau were analyzed for T22 brightness in the first 100 um of every slice. AH36 brightness was normalized to CaMKII.eYFP brightness to control for any potential variability in viral expression. All groups were then normalized to the Ctrl injected condition. hAPP+hTau showed a significantly higher level of T22 brightness, above both hAPP (p=0.0350) and hTau (p=0.0.0389) (df=8, One-Way ANOVA with Multiple Comparisons). hAPP and hTau were not significantly different (p=0.9526).

Beyond hyperexcitability, the LEC is also the first cortical region to develop tau pathology^5,29–32^. Although Alzheimer’s is characterized by early hAPP/Aβ and later Tau pathology, respectively, the relationship between hAPP, hyperexcitability, and Tau remains unclear. It has previously been established that artificially increasing neuronal activity can accelerate tau pathology^33–35^. However, long-term transgene expression of human Tau (hTau) may act to dampen circuit excitability^4,36,37^ (but see^38^). Thus, we sought to assess the interplay of hAPP-induced circuit hyperexcitability and hTau expression in the LEC. To achieve this, we packaged full-length wild-type human Tau (hTau) into a separate AAV to induce Tau expression locally in the entorhinal cortex. Spontaneous post-synaptic currents were then recorded from LII principal cells, 3 weeks after hAPP alone, hTau alone, or hAPP + hTau co-injection. With hAPP, we again observed an elevated E:I frequency ratio (sEPSC frequency/sIPSC frequency, normalized to control) as described earlier (Fig. 6b). We hypothesized that hTau would result in a reduced E:I ratio with respect to the control baseline. Although the E:I ratio with hTau alone was less than hAPP alone, E:I balance surprisingly remained unchanged with respect to control levels (Fig. 6b). However, hAPP + hTau resulted in an intermediate effect, which abolished the hyperexcitable phenotype seen with hAPP alone (Fig. 6b). These results agree with a homeostatic role for Tau in maintaining circuit excitability. Beyond synaptic event frequencies, all other spontaneous event properties (i.e., amplitude) were statistically similar between all groups (Extended Data Fig. 10a,b).

We next assessed whether the moderating effect of hTau on circuit activity came at the cost of increased pathology, using antibodies for pSer202/pThr205 phosphorylated tau (AH36) or oligomeric tau (T22). Both control and hAPP-injected conditions showed low levels of AH36 positivity, likely due to labeling endogenous murine tau (Extended Data Fig. 10). While both hTau and hAPP+hTau induced high levels of AH36-positive staining, it appeared that hTau alone injected mice had mostly somatically located staining. In contrast, hAPP+hTau co-injected mice displayed dendritic-based staining (Fig. 6c) suggesting an interaction with hAPP which promotes Tau translocation in entorhinal neurons. Oligomeric tau (T22) (Fig. 6e), which has recently been shown in human tissue as a tau species that likely spreads transsynaptically from axons to other regions^39^, displayed a surprisingly robust increase, but only when hAPP+hTau were co-expressed. Thus, it appears that co-expression of human Tau can restore human APP-induced circuit hyperexcitability but consequentially results in increases in known pathological tau species.

## Discussion

Here we demonstrate that PV interneurons within the LEC are biophysically distinct from other neocortical PV interneurons. Furthermore, differences in the native-state proteomes of PV interneurons from the LEC and SS Ctx regions were marked. Although the WT PV firing frequency in our LEC recordings is consistent with previous obervations^17^, the striking biophysical differences (i.e., AP waveform) with respect to PV cells in other cortical regions had not been systematically evaluated. Together, these cell-specific, regional differences likely underlie differential susceptibility to hAPP-induced pathophysiology. Interestingly, compared to SS, we found that PV interneurons residing in the LEC were significantly enriched in proteins associated with cognitive resilience in humans. A preferential loss of LEC PV interneuron integrity in humans with AD may potentially explain this association. Further studies at the single-cell level in humans with early-stage AD will be necessary to confirm this assertion.

Recent work shows that APP expression moves outside of normal homeostatic levels in models of late-onset AD risk alleles^40,41^. The ratio of different APP isoforms also shifts in human AD, from mainly APP 695 to increasing levels of APP 770 and 751^24,25^. These longer isoforms show increased expression following aging-related processes (e.g., after reproductive hormonal production decline^42^, hypercholesterolemia^43^, and atherosclerosis^44^), all of which are also associated with increased AD risk^45–48^. Thus, here we induced adult-onset expression of hAPP 770 to model these phenomena. We found that shortly after hAPP expression (2–3 weeks), LEC PV interneuron firing became severely disrupted. Interestingly, this hAPP-induced pathophysiology could not be recapitulated following expression of the full-length mouse mAPP gene analogue. Of the 26 amino acids differentiating our hAPP and mAPP proteins, only 3 are situated within the amyloid-beta region. Of note, of the ‘wild-type’ versions of newly designed hAPP knock-in mouse models^49,50^ now in wide use, only the 3 amino acids within the amyloid region are humanized. Further investigation to uncover whether amino acid differences residing solely within the amyloid region are sufficient to perturb LEC PV interneuron function, as described here.

GABAergic interneurons require homeostatic APP levels for proper physiological function and circuit activity control^51^. Furthermore, APP^52^, as well its cleavage proteins^53–55^ and products^56,57^, can modulate neuronal biophysics and alter the expression of ion channels, many of which are essential for maintaining the ‘fast-spiking’ phenotype of PV interneurons. Modifications to Na_v_1 or K_v_3 channel availability in different constitutive hAPP-expressing mice have recently been linked to reduced PV excitability^9,15^. Although short-term hAPP expression in this study could significantly reduce PV firing in LEC, we observed no biophysical indicators implicating changes to either Na_v_1 or K_v_3 availability. Thus, alternative biophysical mechanisms must be responsible for our observations following more short-term hAPP expression in adult mice. Notably, we observed a substantial decrease in input resistance in LEC PV cells expressing hAPP. This could be due to enhanced availability of leak channels or potentially low-voltage activating K^+^ conductances, such as KCNQ (K_v_7), which curiously have been shown to be regulated by APP cleaving proteins^53^ and cleavage products^56,58^. We cannot rule out that longer hAPP expression times *in vivo* may induce other changes through distinct pathological or homeostatic processes. Further dissection of hAPP, its cleavage products, and accompanying proteins over different time scales will be necessary to further understand mechanisms of PV and excitatory cell dysfunction.

The LEC is also the first cortical region to develop tau pathology^5,29–32^. Yet, the relationship between hAPP, hyperexcitability, and Tau remains unclear. It has previously been established that artificially increasing neuronal activity can accelerate tau pathology^33–35^. However, the expression of hTau has been suggested to strongly dampen circuit excitability^4,36,37^ (but see^38^). Here we observed that hTau co-expressed with hAPP results in an intermediate circuit excitability level when compared to hAPP or hTau injected alone. Trans-synaptic spread of tau has been shown from the entorhinal cortex to other brain regions^59,60^, and most recently this spread has been suggested to occur in human patients via the oligomeric tau species (T22+)^39^. Remarkably, here we show that although hTau co-injection with hAPP normalized circuit excitability, it also caused a significant increase in this oligomeric tau species. Further research is necessary to determine if this resultant oligomeric species displays a similar trans-synaptic spread to downstream regions, such as the dentate gyrus.

The LEC is the first cortical region to undergo end-stage cellular neurodegeneration^5^ in AD, specifically, Layer II^2^ excitatory cells^7^. Conversely, one of the earliest pathophysiological alterations seen in both humans with AD, and in mouse models of early- and late-onset AD pathology^3,4,11^ is altered local circuit excitability^6,61,62^. In agreement with our *ex vivo* mechanistic cellular findings here, hyperactivity has been shown to preferentially emerge in the LEC region *in vivo^6^*. Our study suggests that hAPP-induced hyperexcitability in the LEC arises not from alterations in the intrinsic or synaptic properties of AD-vulnerable LII excitatory cells, but rather from an initial alteration in intrinsic excitability of surrounding PV interneurons. The fact that short-term hAPP expression in SS cortex caused no changes in PV firing or overall basal circuit excitability also supports this notion. Circuit hyperexcitability is likely an influential factor in the neurodegenerative cascade, as it has been shown to exacerbate release of amyloid-beta^63^, and also promotes tau pathology and subsequent trans-synaptic tau spreading^34^, which ultimately induces spine degeneration^16^ and cell death^64^. Ultimately, regions that first undergo hyperexcitability may also be among the earliest to display these pathological markers as the disease progresses^34,65^.

## Methods

### Acute slice preparation

All animal procedures were approved by the Emory University IACUC. Acute slices from SS cortex and LEC were prepared from a mixture of C57Bl/6J and PV-Cre mice (8–12 weeks old), evenly dispersed between test and control groups. Male and female mice were used for all experiments with data collected from ≥ 3 mice per experimental condition. Mice were first anesthetized and then killed by decapitation. The brain was then immediately removed by dissection in ice-cold cutting solution (in mM) 87 NaCl, 25 NaHO3, 2.5 KCl, 1.25 NaH2PO4, 7 MgCl2, 0.5 CaCl2, 10 glucose, and 7 sucrose. Brain slices (250 μm) were sectioned in the sagittal plane using a vibrating blade microtome (VT1200S, Leica Biosystems) in the same solution. Slices were transferred to an incubation chamber and maintained at 34°C for ~30 min and then at 23–24°C thereafter. During whole-cell recordings, slices were continuously perfused with (in mM) 128 NaCl, 26.2 NaHO3, 2.5 KCl, 1 NaH2PO4, 1.5 CaCl2, 1.5MgCl2 and 11 glucose, maintained at 30.0±0.5°C. All solutions were equilibrated and maintained with carbogen gas (95% O2/5% CO2) throughout.

### Electrophysiology

PV interneurons and excitatory cells were targeted for somatic whole-cell recording in layer 5 region of somatosensory cortex or layer 2 lateral entorhinal cortex by combining gradient-contrast video-microscopy with epifluorescent illumination on custom-built or commercial (Olympus) upright microscopes. Electrophysiological recordings were acquired using Multiclamp 700B amplifiers (Molecular Devices). Signals were filtered at 10 kHz and sampled at 50 kHz with the Digidata 1440B digitizer (Molecular Devices). For whole cell recordings, borosilicate patch pipettes were filled with an intracellular solution containing (in mM) 124 potassium gluconate, 2 KCl, 9 HEPES, 4 MgCl2, 4 NaATP, 3 L-Ascorbic Acid and 0.5 NaGTP. For experiments recording spontaneous and miniature events, an intracellular solution containing (in mM) 120 CsMeSO4, 10 HEPES, 5 TEA.Cl, 4 Na2ATP, 0.5 Na2GTP, 2 MgCl2, 10 L-Ascorbic Acid, and 3 Qx314. To obtain miniature events, slices were perfused with 1 μM TTX for 10 minutes prior to recording (at least 10 mL run). The same protocol was used for spontaneous and miniature events. Pipette capacitance was neutralized in all recordings and electrode series resistance compensated using bridge balance in current clamp. Liquid junction potentials were uncorrected. Recordings had a series resistance < 20 MΩ. Membrane potentials were maintained near −70 mV during current clamp recordings using constant current bias. Action potential trains were initiated by somatic current injection (300 ms) normalized to the cellular capacitance in each recording measured immediately in voltage clamp after breakthrough^66^. For quantification of individual AP parameters, the 1^st^ AP in a spike train at was analyzed at 12 pA/pF for all cells. Passive properties were determined by averaging the responses of several 100 ms long, −20pA steps during each recording. Spontaneous and miniature events were recorded at a holding voltage of −70 and 0 mV, one second each, interleaved for 3–5 minutes. For regional comparisons of PV interneurons, combined controls from datasets in each region were used. Event detection was carried out using Clampfit (Molecular Devices) using a template matching algorithm and were curated manually with a 4 kHz low-pass filter.

### Intracranial viral injections:

5–9 week old mice were injected with AAV(PHP.eB).E2.tdTom with saline or AAV(PHP.eB).EF1a.hAPP (0.3 μL total, 1:1) in the SBFI vibrissal region of cortex or the Lateral Entorhinal Cortex. For murine APP experiments AAV(PHP.eB).EF1a.hAPP was replaced with AAV(PHP.eB).EF1a.mAPP. For tau experiments, the four conditions were: Ctrl (CaMKII.eYFP: saline 1:2), hAPP (EF1a.hAPP: CaMKII.eYFP: saline, 1:1:1), hTau (AAV(PHP.eB).EF1a.hMAPT: CaMKII.eYFP: saline, 1:1:1), and hAPP+hTau (EF1a.hAPP: Ef1a.hMAPT: CaMKII.eYFP, 1:1:1). When performing intracranial viral injections, mice were head-fixed in a stereotactic platform (David Kopf Instruments) using ear bars, while under isoflurane anesthesia (1.5 – 2.0%). Thermoregulation was provided by a heating plate using a rectal thermocouple for biofeedback, thus maintaining core body temperature near 37°C. Bupivacaine was subcutaneously injected into the scalp to induce local anesthesia. A small incision was opened 5–10 minutes thereafter and a craniotomy was cut in the skull (< 0.5 μm in diameter) to allow access for the glass microinjection pipette. Coordinates (in mm from Bregma) for microinjection in the SS Cortex were X = ± 0.85; Y = −2.5; α = 0°; Z = −0.85, coordinates for the LEC were X = ± 3.39; Y = −4.52; α = 0°, Z= −2.4, −1.5. Viral solution (titer 1×10^09^ to 1×10^13^ vg/mL) was injected slowly (~0.02 μL min-1) by using a Picospritzer (0.3 μL total). After ejection of virus, the micropipette was held in place (5 min) before withdrawal. The scalp was closed with surgical sutures and Vetbond (3M) tissue adhesive and the animal was allowed to recover under analgesia provided by injection of carprofen and buprenorphine SR. After allowing for onset of expression, animals were sacrificed acute slices were harvested.

### Retro-orbital (RO) injection:

Male and female mice were given AAV retro-orbital injections as previously described^67^. Mice were anesthetized with 1.8–2% isoflurane. AAV(PHP.eB).E2.Cre.2A.GFP virus was titrated to 2.4×10^11^ vector genomes total was accompanied by AAV(PHP.eB).Flex.tdTom titrated to 3.1×10^11^ and injected in TurboID+ mice^20^ to label PV interneurons throughout cortex. Titrated virus was injected into the retro-orbital sinus of the left eye with a 31G × 5/16 TW needle on a 3/10 mL insulin syringe. Mice were kept on a heating pad for the duration of the procedure until recovery and then returned to their home cage. After 3 weeks post-injection, mice were provided with biotin water continuously. Biotin water was administered for 2 weeks until acute slice sample collection (total of 5 weeks post-RO injection).

### CIBOP studies:

PV-CIBOP studies were performed by single retro-orbital injections of AAV(AAV(PHP.eB).E2.Cre.2A.GFP) as described above to Rosa26TurboID/wt mice and WT littermate animals at 7 weeks of age, as previously described^21^. Control groups in PV-CIBOP studies also received AAV E2.Cre injections for fair comparisons. After 3 weeks to allow Cre-mediated recombination and TurboID expression, biotinylation (37.5 mg/L in drinking water) was performed for 2 weeks^20^. After 5 weeks (total), acute slices were acquired as described above, with subsequent microdissection of the SS Ctx or the LEC.

### Tissue processing for protein-based analysis, including Western Blot (WB):

Tissue processing for proteomic studies, including Mass Spectrometry (MS), were performed similarly to previous CIBOP studies^20,21^. Frozen brain tissues (whole brain homogenate excluding cerebellum for WB and microdissected cortical regions for Fig 1) either intact or dissected cortex, was weighed and added to 1.5mL Rino tubes (Next Advance) containing stainless-steel beads (0.9–2mm in diameter) and six volumes of the tissue weight in urea lysis buffer (8 M urea, 10 mM Tris, 100 mM NaH2PO4, pH 8.5) containing 1X HALT protease inhibitor cocktail without EDTA (78425, ThermoFisher). Tissues were homogenized in a Bullet Blender (Next Advance) twice for 5 min cycles at 4 °C. Tissue were further sonicated consisting of 5 seconds of active sonication at 20% amplitude with 5 seconds incubation periods on ice. Homogenates were let sit for 10 minutes on ice and then centrifuged for 5 min at 12,000 RPM and the supernatants were transferred to a new tube. Protein concentration was determined by BCA assay using Pierce^™^ BCA Protein Assay Kit (23225, Thermofisher scientific). For WB analyses, 10μg of protein from brain lysates were used to verify TurboID expression (anti-V5) and biotinylation (streptavidin fluorophore conjugate). Standard WB protocols, as previously published, were followed^20^. All blots were imaged using Odyssey Infrared Imaging System (LI-COR Biosciences) or by ChemiDoc Imaging System (Bio-Rad) and densitometry was performed using ImageJ software.

### Enrichment of biotinylated proteins from CIBOP brain:

As per CIBOP protocols previously optimized^20^, biotinylated proteins were captured by streptavidin magnetic beads (88817; Thermofisher Scientific) in 1.5 mL Eppendorf LoBind tubes using 83uL beads per 1mg of protein (here 200 μg of protein with 16.6 μL of beads) in a 500 μL RIPA lysis buffer (RLB)(50 mM Tris, 150 mM NaCl, 0.1% SDS, 0.5% sodium deoxycholate, 1% Triton X-100). In brief, the beads 16.6 μL beads were washed twice with 1 ml of RLB and 200μg of protein were incubated by making up the total volume of the solution up to 500 μl using RPL. After incubation at 4 deg C for 1 h with rotation, beads were serially washed at room temperature (twice with 1 mL RIPA lysis buffer for 8 min, once with 1 mL 1 M KCl for 8 min, once with 1 mL 0.1 M sodium carbonate (Na2CO3) for ~10 s, once with 1 mL 2 M urea in 10 mM Tris-HCl (pH 8.0) for ~10 s, and twice with 1 mL RIPA lysis buffer for 8 min), followed by 1 RIPA lysis buffer wash 4 final PBS washes. Finally, after placing the tubes on the magnetic rack, PBS was removed completely, then the beads were further diluted in 100 μl of PBS. The beads were mixed and 10% of this biotinylated protein coated beads were used for quality control studies to verify enrichment of biotinylated proteins (including WB and silver stain of proteins eluted from the beads). Elution of biotinylated protein was performed by heating the beads in 30 μL of 2X protein loading buffer (1610737; BioRad) supplemented with 2 mM biotin + 20 mM dithiothreitol (DTT) at 95°C for 10 min. The remaining 90% of sample were stored at −20°C for western blot or mass spectrometric analysis of biotinylated protein.

### Western blotting and Silver Stain:

To confirm protein enrichment on the pulldown samples the protein were eluted from the beads as mentioned above and 1/3^rd^ of the eluted protein was resolved on a 4–12% Bris-Tris gel (Invitrogen: cat# NW04125box) and transferred onto transferred onto iBlot 2 Transfer Stack containing nitrocellulose membrane using the BOLT transfer system. The membranes were washed once with TBS-T (0.1% tween-20) and then blocked with Start Block (37543, Thermofisher Scientific) for 1 h. The membrane was then probed with streptavidin-Alexa-680 diluted in Start Block for 1 h at room temperature. Further, the membranes were washed 3 times and biotinylated proteins were detected on Odyssey Infrared Imaging System (LI-COR Biosciences). In parallel, 2/3^rd^ of the eluted protein samples were resolved on a 4–12% Bris-Tris gel (Invitrogen: cat# NW04125box) and subjected to Silverstein (Pierce™ Silver Stain Kit: Catalog number: 24612) to detect the total amount of protein in each lane according to the manufactured protocol.

### Protein digestion, MS, protein identification and quantification:

On-bead digestion of proteins (including reduction, alkylation followed by enzymatic digestion by Trypsin and Lys-C) from SA-enriched pulldown samples (1mg protein used as input) and digestion of bulk brain (input) samples (25 μg protein), were performed as previously described with no protocol alterations^20,21^. In brief, after the removal of PBS from remaining 90% of streptavidin beads (10% used for quality control using western blot and silver stain) were resuspended in 90 uL of 50 mM ammonium bicarbonate (NH4HCO3) buffer. Biotinylated proteins were then reduced with 1 mM DTT and further alkylated with 5 mM iodoacetamide (IAA) in the dark for 30 min each on shaker. Proteins were digested overnight with 0.2 μg of lysyl (Lys-C) endopeptidase (127–06621; Wako) at RT on shaker followed by further overnight digestion with 0.4 μg trypsin (90058; ThermoFisher Scientific) at RT on shaker. The resulting peptide solutions were acidified to a final concentration of 1% formic acid (FA) and 0.1% triflouroacetic acid (TFA), desalted with a HLB columns (Cat#186003908; Waters). The resulting protein solution was dried in a vacuum centrifuge (SpeedVac Vacuum Concentrator). Detailed methods for this protocol have been previously published^20^. Lyophilized peptides were resuspended followed by liquid chromatography and MS (Q-Exactive Plus, Thermo, data dependent acquisition mode) as per previously published protocols^20^. As previously published^68–70^, MS raw data files were searched using SEQUEST, integrated into Proteome Discoverer (ThermoFisher Scientific, version 2.5) using the Uniprot 2020 database as reference (91,441 target 37 sequences including V5-TurboID). Raw MS data as well as searched Proteome Discoverer data before and after processing to handle missing values, will be uploaded to the ProteomeXchange Consortium via the PRIDE repository^71^. The false discovery rate (FDR) for peptide spectral matches, proteins, and site decoy fraction were 1 %. Other quantification settings were similar to prior CIBOP studies^20^. Quantitation of proteins was performed using summed peptide abundances given by Proteome Discoverer. We used razor plus unique peptides for protein level quantitation. The Proteome Discoverer output data were uploaded into Perseus (Version 1.6.15) and abundance values were log2 transformed, after which data were filtered so that >50% of samples in a given CIBOP group expected to contain biotinylated proteins, were non-missing values. Protein intensities from SA-enriched pulldown samples (expected to have biotinylated proteins by TurboID) were normalized to sum column intensities prior to comparisons across groups. This was done to account for any variability in level of biotinylation as a result of variable Cre-mediated recombination, TurboID expression and/or biotinylation^20^.

### RNAscope:

RNAscope was performed for confirmation of viral hAPP and mAPP expression, as well as quantification of viral expression in a cell-type-specific manner. RNAscope was performed as instructed by Advanced Cell Diagnostics (ACD). Tissue was prepared from C57Bl/6J mice (8–12 weeks old), evenly dispersed between test and control groups. Male and female mice were used for all experiments with data collected from ≥ 3 mice per experimental condition. Mice were injected with AAV(PHP.eB).E2.tdTom and AAV(PHP.eB).hAPP or AAV(PHP.eB).mAPP (0.3 uL total, 1:1) in one hemisphere and injected contralaterally with AAV(PHP.eB).E2.tdTom and saline (0.3 uL total, 1:1), n=3 each. Hemispheres were randomized. After 2–3 weeks, mice were first anesthetized and then killed by decapitation. The brain was then immediately removed and flash frozen in isopentane on dry ice. Samples were kept at −80°C prior to sectioning. Tissue was sectioned on a cryostat at 16 μm thickness, mounted onto Superfrost Plus Slides, and stored at −80°C until use. Samples were fixed and dehydrated according to the RNAscope kit manufacturer (ACDBio) standard protocol. In brief, frozen slides containing tissue sections were immediately dipped in pre-chilled 4% paraformaldehyde (PFA) for 15 minutes at room temperature. After fixation, slides were briefly rinsed with 1× phosphate buffered saline (PBS) two times to remove excess fixative. Tissue sections were then dehydrated in a series of ethanol solution 50%, 70% and 100% for 5 min at room temperature. After ethanol washes the slides were transferred into a fresh 100% ethanol solution to sit overnight at −20°C. The following day, slides were taken out of the ethanol solution, air dried for 5 minutes, and hydrophobic barriers were drawn around each section. The remainder of the RNAscope assay was then performed following the manufacturer’s protocol, multiplexing two different probe groups on two different sections from each animal: 1) human APP (catalog #418321), mouse APP (catalog #519001-C2), and CaMKIIα (catalog #445231-C3), or 2) human APP, mouse APP, and Pvalb (catalog #421931-C3).

### Immunohistochemistry:

Acute slices were acquired as previously described at 100 μm thickness. Immediately following collection from the vibratome, free-floating sections were placed in 4% paraformaldehyde for fixation at room temperature for 1–2 hours. Sections were then washed three times in 1x Tris Buffered Saline (1xTBS) for 10 minutes. To block nonspecific binding, the sections were then incubated with 5% goat serum (in 1xTBS) for 1 hour at room temperature. Sections were then incubated overnight at 4°C on a shaker plate in the primary antibody solution, which contained 0.2% Triton X-100, 1% goat serum, and a 1:1000 dilution of either the AH36 antibody (StressMarq Sciences, #SMC-601) or T22 antibody (Millipore Sigma, #ABN454) in 1xTBS. The next day, sections were washed three times for 10 minutes in 1xTBS before incubation with the secondary antibody (Alexa Fluor^™^ 647 at 1:1000; ThermoFisher Scientific, #A-21245) in 1xTBS for 1 hour at room temperature on a shaker plate. From the point of secondary antibody incubation, sections were protected from light using aluminum foil. Following secondary antibody incubation, sections were washed again three times for 10 minutes in 1xTBS, mounted on slides (Fisher Scientific, #1255015), and coverslipped with Fluoromount containing DAPI (ThermoFisher Scientific, #00–4959-52). Slides were then imaged on a Nikon C2 laser-scanning confocal system with an inverted Nikon ECLIPSE Ti2 microscope. Imaging parameters (e.g., laser power, gain) were defined for each primary antibody and were kept consistent between all sections in that primary antibody group.

### Image Analysis:

Images for analysis of RNAscope sections were taken on a Keyence BZ-X800 microscope (KEYENCE; Osaka, Japan) at 40X magnification. Two images were acquired of each mouse LEC hemisphere, and 4 sections were imaged per mouse (total: ~8 images/experiment for an n=3). The acquisition parameters were kept constant throughout imaging of all sections. Four fluorescent channels were used simultaneously; (1) the green channel was assigned for VIVID 520 dye (human APP probe), (2) the blue channel was used for DAPI nuclear stain, (3) the red channel was assigned for VIVID 570 dye (mouse APP probe), and (4) the far-red channel was assigned for VIVID 650 dye (CaMKIIα or Pvalb probes). A z-stack was taken (with 1 μm steps) of each hemisphere, and the full focus feature in the Keyence BZ-X800 analysis software was applied to compress each 10 μm z-stack. These compressed z-stacks were then used for image analysis in HALO v3.6 FISH-IF v2.1.4 (Indica Labs). For immunohistochemistry experiments, 2 sections were imaged per antibody per mouse (total: ~4 images/experiment for an n=3–5). The acquisition parameters were kept constant throughout imaging of all sections. Two channels were used simultaneously; (1) the green channel was assigned for CaMKII.eYFP expression, (2) the red channel was assigned for Alexa Fluor™ 647. For each slice, four line scans of 200 thickness were analyze from the pia to the end of the 60x photo (~180–200 μm total) using ImageJ software. Antibody brightness was normalized to CaMKII.eYFP expression for each condition to control for any slight variation in viral expression. The obtained data was then analyzed for figure generation in Prism (GraphPad). The process from sample fixation to image analysis covered a four-day time frame.

### Flow Cytometry:

Flow cytometry was performed for the confirmation of viral hAPP protein expression. For positive control 5xFAD and their wild-type littermates were used and injected stereotactically in the somatosensory cortex with 1:1 ratio of AAV(PHP.eB).E2.GFP and saline (0.3 μL). While for hAPP expression C57Bl/6J were also stereotactically injected at a 1:1 ratio with AAV(PHP.eB).E2.GFP and saline or AAV(PHP.eB).hAPP (0.3 μL total). 2–3 weeks after the injections, mice were euthanized (8–10 weeks old) by decapitation and acute slices of 250 μm were obtained and then micro-dissected to isolate the cortical region containing GFP^+^ expressing cells using an epi-fluorescent stereoscope (Olympus SZX12). Virus-injected microdisected regions of the brain were then placed into a cutting solution with 0.5 mg/mL protease (P5147–100MG, Sigma-Aldrich) for 60 minutes with continuous carbogen gas bubbling. Samples were then manually triturated in 300 μL of 1% PBS into a single-cell suspension. Cells were stained with a human-specific APP antibody (SIG-39320, Biolegend). Cells were first fixed in 1x fixation buffer (eBioscience; cat# 00–8222-49) for 30 min on ice, then washed 3x in PBS. Cells were then permeabilized for 30 min using 1x permeabilization buffer (eBioscience; cat# 00–8333-56) on ice. To determine the presence of hAPP in these cells, fixed and permeabilized cells were incubated with human-specific APP antibody (SIG-39320, Biolegend) at dilution 1:250 for 1 h. cells were then washed with permeabilization buffer 3 times and incubated for 30 minutes with a secondary antibody at dilution 1:500. Cells were finally washed 3 times with permeabilization buffer, as mentioned above. After the last wash, 250 μl of PBS was added, vortexed and kept on ice in the dark until flow cytometry was performed. Compensation was performed prior to the experiment using a single fluorophore labelled OneComp eBeads^™^ Compensation Beads (Catalog number: 01–1111-41; Thermofisher).

### Stats and Analysis

Custom python scripts, Axograph, Graphpad Prism (Graphpad Software), and Excel (Microsoft) were used for analysis with values in text and figures. Statistical differences were deemed significant with α values of p < 0.05. Two-tailed unpaired and paired t-tests were used for unmatched and matched parametric datasets, respectively. Where appropriate, group data were compared with 1 or 2-way ANOVA and significance between groups noted in figures was determined with Tukey’s or Sidak’s multiple post-hoc comparison tests. Normality was determined using D’Agostino & Pearson omnibus or Shapiro-Wilk tests.

### K-means clustering and Principal Component Analysis:

K-means clustering and Principal component analysis (PCA) were conducted on datasets from excitatory neurons and PV interneurons, respectively, in the LEC. All passive and active properties were used for each cell to conduct unsupervised clustering. Post-clustering and analysis, Ctrl or hAPP identities were restored to their respective cells.

### Analyses of MS data and bioinformatics analyses:

Within each MS study, we compared bulk proteomes to SA-enriched proteomes to confirm that expected proteins (from either PV-INTs) were indeed enriched while nonneuronal proteins (e.g. glial proteins) were de-enriched as compared to bulk brain proteomes. We also identified proteins unique to bulk or SA-enriched pulldown samples. Within SA-enriched biotinylated proteins, we restricted our analyses to those proteins that were confidently biotinylated and enriched (based on statistical significance unadj. P<0.05 as well as 2-fold enrichment in biotinylated vs. non biotinylated samples). This allowed us to exclude proteins that were non-specifically enriched by streptavidin beads. Within biotinylated proteins, group comparisons were performed using a combination of approaches, including differential abundance analysis, hierarchical clustering analysis (Broad Institute, Morpheus, https://software.broadinstitute.org/morpheus), as well as PCA, (in SPSS Ver 26.0 or R). Differential abundance analyses were performed on log2 transformed and normalized abundance values using two-tailed unpaired T-test for 2 groups assuming equal variance across groups or one-way ANOVA + post-hoc Tukey HSD tests for >2 groups). Unadjusted and FDR-corrected comparisons were performed, although we relied on unadjusted p-values along with effect size (fold-enrichment) to improve stringency of analyses. After curating lists of differentially enriched proteins, gene set enrichment analyses (GSEA) were performed (AltAnalyze Ver 2.1.4.3) using all proteins identified across bulk and pulldown proteomes as the reference (background list). Protein-protein-interactions between proteins within lists of interest were examined using STRING (https://stringdb.org/cgi/input?sessionId=bqsnbjruDXP6&input_page_show_search=on)123. We also performed GSVA of DEPs identified in bulk as well as PV-IN proteomes from SS Ctx and LEC to complement GSEA^72,73^. As previously published, statistical differences in enrichment scores for each ontology comparing two groups, were computed by comparing the true differences in means against a null distribution which was obtained by 1000 random permutations of gene labels. Benjamini & Hochberg false discovery rate adjusted p values <0.05 were considered significant. The reference gene sets for GSVA were the M5 (Mouse) Ontology Gene Sets from MSigDB (https://www.gseamsigdb.org/gsea/msigdb/mouse/collections.jsp?targetSpeciesDB=Mouse#M5).

## Extended Data

**Extended Data Figure 2.**
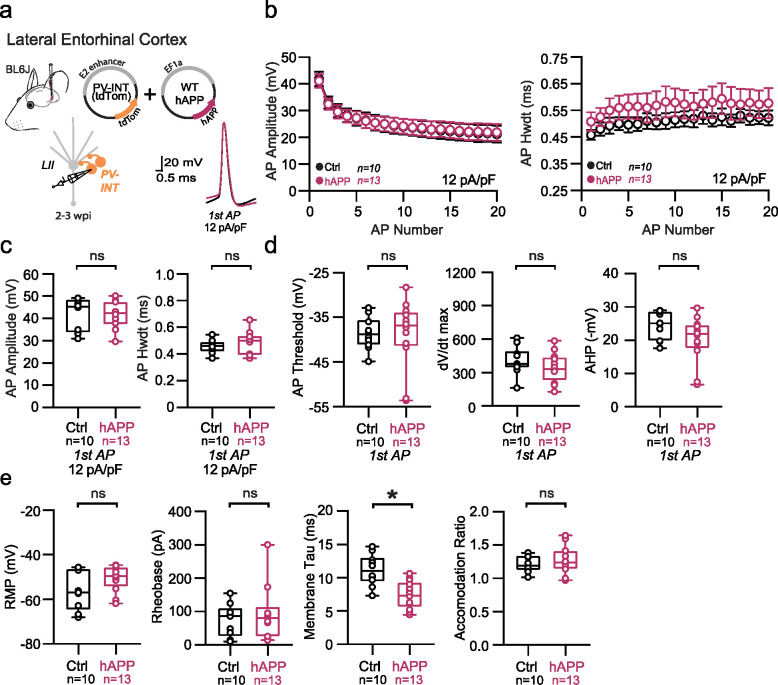
Passive and active properties of LEC PV interneurons after hAPP injection **a.** Graphical summary of AAV.E2.tdTom and AAV.EF1a.hAPP (or for Ctrl, saline) stereotactic injection in the Lateral Entorhinal Cortex. tdTom+ PV interneurons were fluorescently targeted for whole-cell current-clamp recordings. AP waveforms of tdTom+ PV interneurons were compared at 12 pA/pF square pulse injections in WT mice from Ctrl and hAPP injected. Aps from the 1^st^ spike in the train are superimposed for comparison. **b.** Relationship between AP amplitude (p=0.8848, df=21) or width (p<0.0001, df=21) in WT mice and AP # during spike trains elicited with a 12 pA/pF current injection. **c.** Summary data of AP properties. LEC PV interneurons after hAPP injection 1^st^ AP amplitude (p=0.9365, t=0.0809) and half-width remains unchanged (p=0.5096, t=0.6746, df=21). **d.** Summary data of AP properties. LEC PV interneurons after hAPP injection AP threshold (p=0.8471, t=0.1964), dV/dt max (p=0.1228, t=1.624), and AHP (0.6284, t=0.4929) (df=21) remained unchanged. **e.** Summary data of AP properties. LEC PV interneurons after hAPP injection show unchanged Resting Membrane Potential (p=0.0898, t=1.788), Rheobase (p=0.0516, t=2.070) and Accomodation Ratio (p=0.6616, t=0.4443), but a reduction in Membrane Tau(p=0.0008, t=3.933) (df=21). For all summary graphs, data are expressed as mean (± SEM). For **b**: Statistical significance is denoted as *=p<0.05, as determined by Two-way ANOVA with Sidak’s multiple comparison test. For c, d, e: Individual data points and box plots are displayed. Statistical significance is denoted as *=p<0.05, as determined by two-tailed unpaired t-test.

**Extended Data Figure 3.**
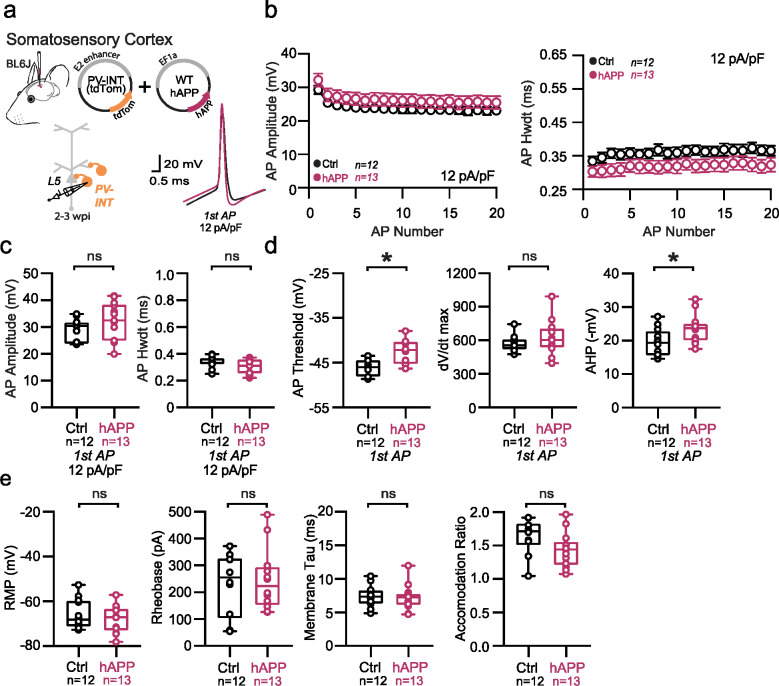
Passive and active properties of SS Ctx interneurons after hAPP injection **a.** Graphical summary of AAV.E2.tdTom and AAV.EF1a.hAPP (or for Ctrl, saline) stereotactic injection in the SS Cortex. tdTom+ PV interneurons were fluorescently targeted for whole-cell current-clamp recordings. AP waveforms of tdTom+ PV interneurons were compared at 12 pA/pF square pulse injections in WT mice from Ctrl and hAPP injected. Aps from the 1^st^ spike in the train are superimposed for comparison. **b.** Relationship between AP amplitude (p=0.1688, t=1.423 df=23) or width (p=0.1506, t=1.489 df=23) for in WT mice and AP # during spike trains elicited with a 12 pA/pF current injection. **c.** Summary data of AP properties. SS PV interneurons after hAPP injection 1^st^ AP amplitude (p=0.1688, t=1.423) and half-width (p=0.1506, t=1.489) (df=23) remain unchanged. **d.** Summary data of AP properties. SS PV interneurons after hAPP injection dV/dt max remains unchanged (p=0.3229, t=1.011). AP Threshold (p=0.0010, t=3.839) and AHP (p=0.0237, t=2.422)(df=23) significantly increase after hAPP injection. **e.** Summary data of AP properties. SS PV interneurons after hAPP injection show unchanged Resting Membrane Potential (p=0.4043, t=0.8497), Rheobase (p=0.7795, t=0.2838), Accomodation Ratio (p=0.0807, t=1.835), and Membrane Tau (p=0.8098, t=0.2435) (df=23). For all summary graphs, data are expressed as mean (± SEM). For **b**: Statistical significance is denoted as *=p<0.05, as determined by Two-way ANOVA with Sidak’s multiple comparison test. For c, d, e: Individual data points and box plots are displayed. Statistical significance is denoted as *=p<0.05, as determined by two-tailed unpaired t-test.

**Extended Data Figure 4.**
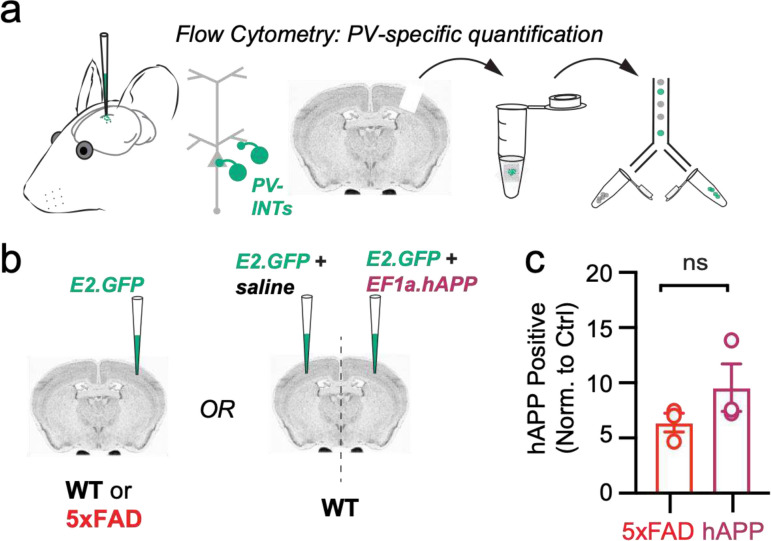
Confirmation of hAPP protein in PV interneurons using PV-specific flow cytometry **a.** Graphical summary of PV-specific flow cytometry workflow. Region containing fluorescent PV interneurons after AAV.E2.GFP stereotactic injection in the SS Cortex was microdissected, triturated, and sorted based on GFP+ signal. Subsequent confirmation specifically for human APP was completed. **b.** WT and 5xFAD mice (~ 2 months) were injected with AAV.E2.GFP and sorted using flow cytometry. WT mice were also used to compare AAV.E2.GFP + EF1a.hAPP injected SS Ctx to the contralateral hemisphere where EF1a was replaced with an equal volume of saline. **c.** Both groups were normalized to their control groups (WT littermates for 5xFAD; contralateral hemi for the hAPP control). The number hAPP expressing PV-interneurons did not significantly differ between 5xFAD and hAPP injected (p=0.2426, t=1.370, df=4).

**Extended Data Figure 5.**
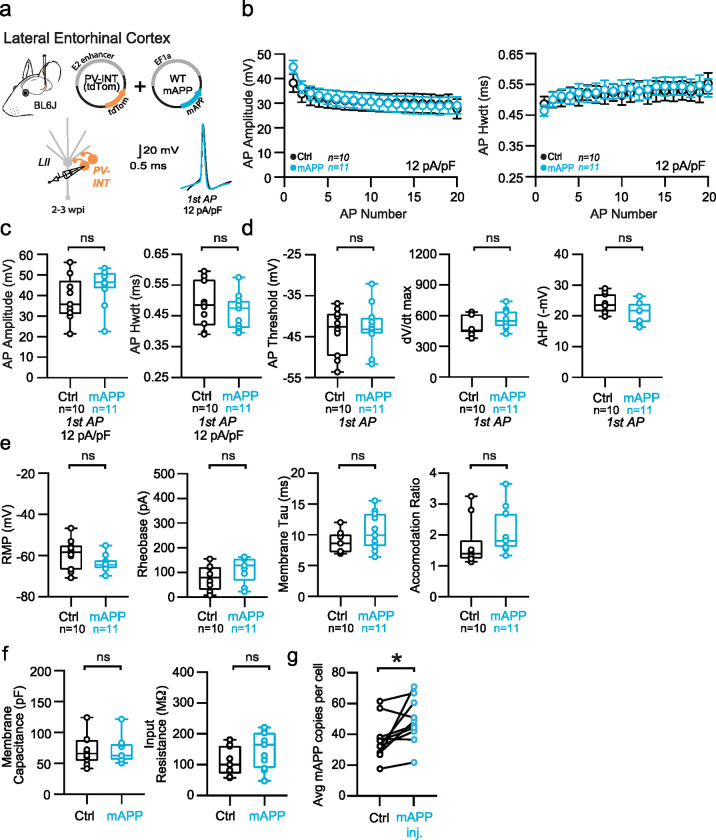
Passive and active properties of LEC PV interneurons after mAPP injection **a.** Graphical summary of AAV.E2.tdTom and AAV.EF1a.mAPP (or for Ctrl, saline) stereotactic injection in the SS Cortex. tdTom+ PV interneurons were fluorescently targeted for whole-cell current-clamp recordings. AP waveforms of tdTom+ PV interneurons were compared at 12 pA/pF square pulse injections in WT mice from Ctrl and mAPP injected. Aps from the 1^st^ spike in the train are superimposed for comparison. **b.** Relationship between AP amplitude (p=0.6057) or width (p=0.3487) in WT mice and AP # during spike trains elicited with a 12 pA/pF current injection. **c.** Summary data of AP properties. LEC PV interneurons after mAPP injection 1^st^ AP amplitude (p=0.1568, t=1.478, df=19) and half-width (p=0.4434, t=0.4434, df=19) remains unchanged. **d.** Summary data of AP properties. LEC PV interneurons after mAPP injection show unchanged AP threshold (p=0.6560, t=0.4526), dV/dt max (p=0.0939, t=1.763), and AHP (p=0.0597, t=2.010)(df=19). **e.** Summary data of AP properties. LEC PV interneurons after mAPP injection show unchanged Resting Membrane Potential (p=0.1147, t=1.657), Rheobase (p=0.1981, t=1.347), Accomodation Ratio (p=0.1688, t=1.431), and Membrane Tau(p=0.1473, t=1.515)(df=19). **f.** Summary data of AP properties. LEC PV interneurons after mAPP injection display an unchanged Membrane Capacitance (p=0.9982, t=0.0023) and input resistance (p=0.1432, t=1.531) (df=19). **g.** RNAscope quantification for mAPP copies per DAPI+ cell comparing mAPP injected to the contralateral hemisphere endogenous mAPP expression. mAPP injected show a significant increase in mAPP copies in the injected hemisphere (p=0.0302, t=2.570, df=9; two-tailed paired t-test). For all summary graphs, data are expressed as mean (± SEM). For **b**: Statistical significance is denoted as *=p<0.05, as determined by Two-way ANOVA with Sidak’s multiple comparison test. For c, d, e, f: Individual data points and box plots are displayed. Statistical significance is denoted as *=p<0.05, as determined by two-tailed unpaired t-test.

**Extended Data Figure 6.**
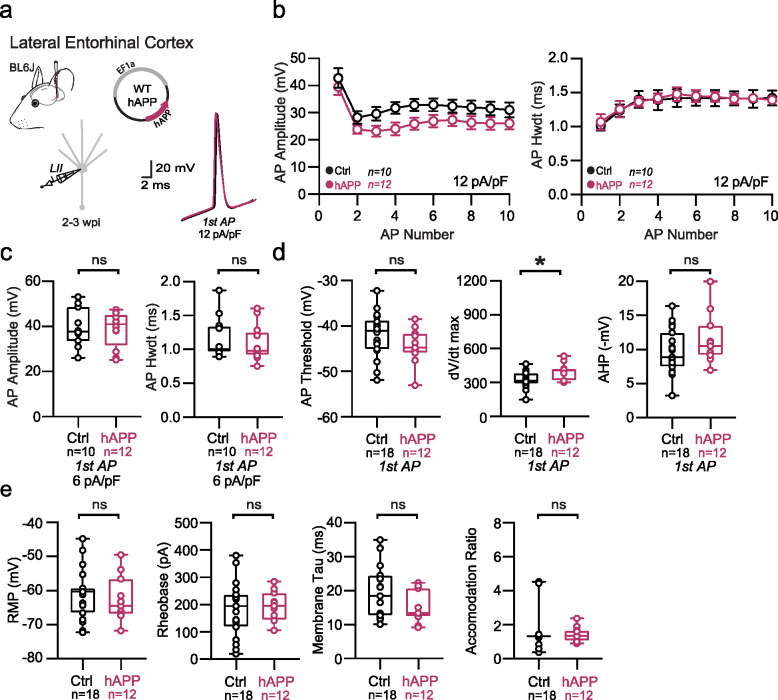
Passive and active properties of LEC excitatory neurons after hAPP injection **a.** Graphical summary of AAV.EF1a.hAPP (or for Ctrl, saline) stereotactic injection in the LEC. Excitatory cells were targeted for whole-cell current-clamp recordings. AP waveforms of excitatory cells were compared at 12 pA/pF square pulse injections in WT mice from Ctrl and hAPP injected. Aps from the 1^st^ spike in the train are superimposed for comparison. **b.** Relationship between AP amplitude or width in WT mice and AP # during spike trains elicited with a 12 pA/pF current injection. **c.** Summary data of AP properties. LEC excitatory cells after hAPP injection 1^st^ AP amplitude (29.36 ± 4.144 mV, 28.62 ± 2.337 mV, hAPP and Ctrl respectively, p=0.8692, t=0.1680, df=28) and half-width remains unchanged (1.257 ± 0.09518 ms, 1.497 ± 0.1281 ms, hAPP and Ctrl respectively, p=0.2255, t=1.278, df=28). **d.** Summary data of AP properties. LEC excitatory cells after hAPP injection AP threshold (p=0.1797, t= 1.377, df = 28) and AHP remain unchanged (p=0.1559, t=1.458, df=28), but dV/dt max shows a significance increase (399.7 ± 21.82, 331.0 ± 16.71), hAPP and Ctrl respectively, p=0.0188, t=2.494, df=28). **e.** Summary data of AP properties. LEC excitatory cells after hAPP injection show unchanged Resting Membrane Potential (p=0.6025, t=0.5267, df=28), Rheobase (p=0.7502, t=3.216, df=28), Accomodation Ratio (p=0.6925, t=0.4001, df=28), and Membrane Tau(p=0.0757, t=1.844, df=28). For all summary graphs, data are expressed as mean (± SEM). For **b**: Statistical significance is denoted as *=p<0.05, as determined by Two-way ANOVA with Sidak’s multiple comparison test. For c, d, e: Individual data points and box plots are displayed. Statistical significance is denoted as *=p<0.05, as determined by two-tailed unpaired t-test.

**Extended Data Figure 7.**
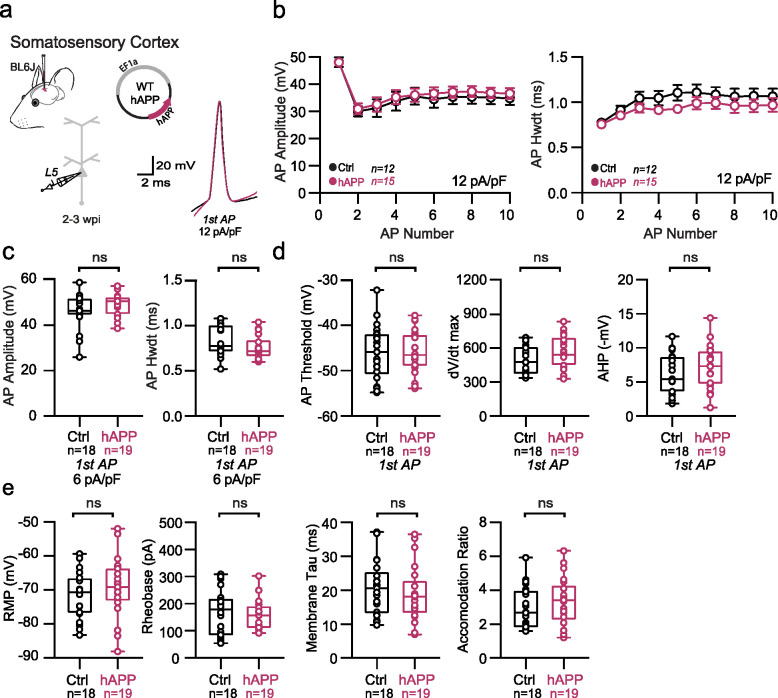
Passive and active properties of SS Ctx excitatory neurons after hAPP injection **a.** Graphical summary of AAV.EF1a.hAPP (or for Ctrl, saline) stereotactic injection in the SS Ctx. Excitatory cells were targeted for whole-cell current-clamp recordings. AP waveforms of excitatory cells were compared at 12 pA/pF square pulse injections in WT mice from Ctrl and hAPP injected. Aps from the 1^st^ spike in the train are superimposed for comparison. **b.** Relationship between AP amplitude or width in WT mice and AP # during spike trains elicited with a 12 pA/pF current injection. **c.** Summary data of AP properties. SS Ctx excitatory cells after hAPP injection 1^st^ AP amplitude (p=0.1884, t=0.1.341) and half-width (p=0.1681, t=1.408) (df=40) remains unchanged. **d.** Summary data of AP properties. SS Ctx excitatory cells after hAPP injection AP threshold (p=0.9682, t=0.04014, df=40),, dV/dt max (p=0.0818, t=1.787, df=40), and AHP remain unchanged (p=0.3229, t=1.001, df=40). **e.** Summary data of AP properties. SS Ctx excitatory cells after hAPP injection show unchanged Resting Membrane Potential (p=0.3725, t=0.9018, df=40), Rheobase (p=0.2159, t=1.258, df=40), Accomodation Ratio (p=0.2538, t=1.158, df=40), and Membrane Tau(p=0.4837, t=0.7070, df=40). For all summary graphs, data are expressed as mean (± SEM). For **b**: Statistical significance is denoted as *=p<0.05, as determined by Two-way ANOVA with Sidak’s multiple comparison test. For c, d, e: Individual data points and box plots are displayed. Statistical significance is denoted as *=p<0.05, as determined by two-tailed unpaired t-test.

**Extended Data Figure 8.**
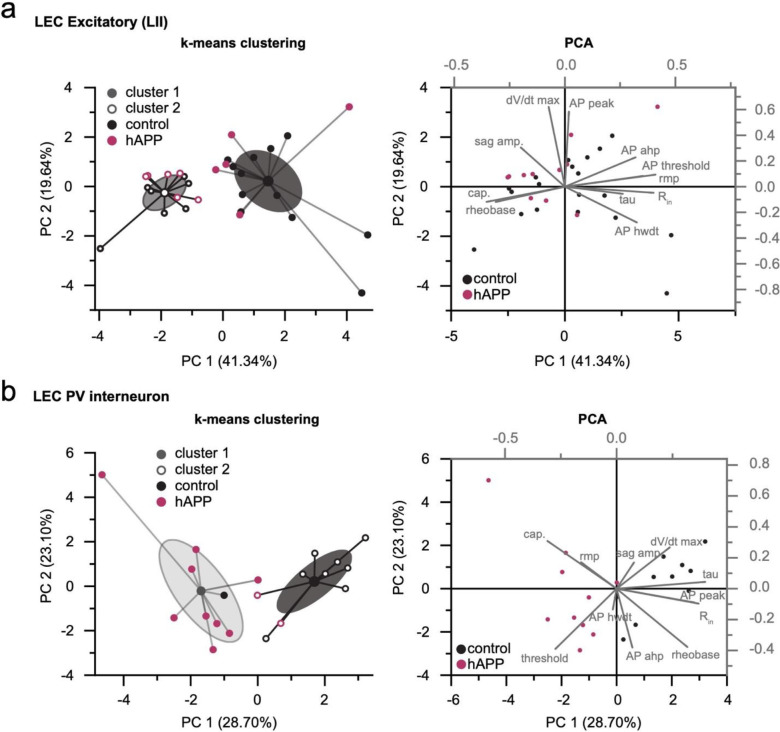
PCA analysis of LEC cell populations after hAPP injection K-means clustering and Principal component analysis (PCA) plot performed on all cells based on active and passive properties for (a) excitatory cells and (b) PV interneurons. a. K-mean clustering fails to separate LEC excitatory cells based on hAPP identity (left), due to largely homogeneous active and passive properties, suggesting differences along different axes. b. Unsupervised clustering preserves hAPP identity in LEC PV interneurons.

**Extended Data Figure 9.**
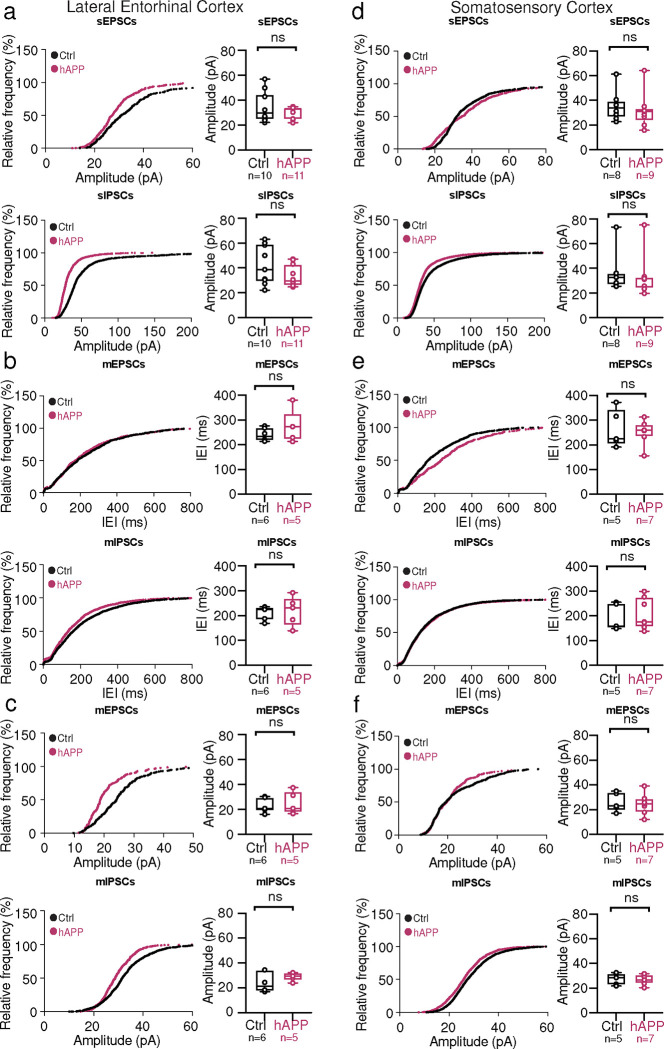
Adult-onset human APP does not alter mini frequencies or event kinetics in the LEC or SS Ctx **a. Top:** Cumulative distribution curve for spontaneous EPSCs in the LEC showing the relationship of relative frequency of events to the amplitude (left). Quantified averages of event amplitude are displayed for each cell as individual data points and compared between Ctrl (black) and hAPP injected (magenta) conditions (right). L2 LEC sEPSCs showed no amplitude change (p=0.3436, t=0.9712, df=19). **Bottom:** Cumulative distribution curve for spontaneous IPSCs in the LEC showing the relationship of relative frequency of events to the amplitude (left). Quantified averages of event amplitude are displayed for each cell as individual data points and compared between Ctrl (black) and hAPP injected (magenta) conditions (right). L2 LEC sIPSCs show an unchanged amplitude (p=0.1144, t=1.659, df=19). **b. Top:** Cumulative distribution curve for miniature EPSCs in the LEC showing the relationship of relative frequency of events to the inter-event interval (left). Quantified averages of event IEI are displayed for each cell as individual data points and compared between Ctrl (black) and hAPP injected (magenta) conditions (right). L2 LEC mEPSCs show no change in the IEI between events (p=0.2765, t=1.158, df=9). **Bottom:** Cumulative distribution curve for miniature IPSCs in the LEC showing the relationship of relative frequency of events to the inter-event interval (left). Quantified averages of event IEI are displayed for each cell as individual data points and compared between Ctrl (black) and hAPP injected (magenta) conditions (right). L2 LEC mIPSCs show no change in the IEI between events (p=0.7963, t=0.2659, df=9). **c. Top:** Cumulative distribution curve for miniature EPSCs in the LEC showing the relationship of relative frequency of events to the amplitude (left). Quantified averages of event amplitude are displayed for each cell as individual data points and compared between Ctrl (black) and hAPP injected (magenta) conditions (right). L2 LEC mEPSCs no amplitude change (p=0.6656, 0.4468, df=9). **Bottom:** Cumulative distribution curve for miniature IPSCs in the LEC showing the relationship of relative frequency of events to the amplitude (left). Quantified averages of event amplitude are displayed for each cell as individual data points and compared between Ctrl (black) and hAPP injected (magenta) conditions (right). L2 LEC mIPSCs show an unchanged amplitude (0.2702, t=1.175, df=9). **d. Top:** Cumulative distribution curve for spontaneous EPSCs in the SS Ctx showing the relationship of relative frequency of events to the amplitude (left). Quantified averages of event amplitude are displayed for each cell as individual data points and compared between Ctrl (black) and hAPP injected (magenta) conditions (right). L5 SS Ctx sEPSCs no amplitude change (p= 0.5669, t=0.5856, df=15). **Bottom:** Cumulative distribution curve for spontaneous IPSCs in the SS Ctx showing the relationship of relative frequency of events to the amplitude (left). Quantified averages of event amplitude are displayed for each cell as individual data points and compared between Ctrl (black) and hAPP injected (magenta) conditions (right). L5 SS Ctx sIPSCs show an unchanged amplitude (p=0.7469, t=0.3297, df=15). **e. Top:** Cumulative distribution curve for miniature EPSCs in the SS Ctx showing the relationship of relative frequency of events to the inter-event interval (left). Quantified averages of event IEI are displayed for each cell as individual data points and compared between Ctrl (black) and hAPP injected (magenta) conditions (right). L2 LEC mEPSCs show no change in the IEI between events (p=0.7372, t=0.3450, df=10). **Bottom:** Cumulative distribution curve for miniature IPSCs in the SS Ctx showing the relationship of relative frequency of events to the inter-event interval (left). Quantified averages of event IEI are displayed for each cell as individual data points and compared between Ctrl (black) and hAPP injected (magenta) conditions (right). L5 SS Ctx mIPSCs show no change in the IEI between events (p=0.6467, t=0.4713, df=10; mEPSC: p=0.7372, t=0.3450, df=10). **f. Top:** Cumulative distribution curve for miniature EPSCs in the SS Ctx showing the relationship of relative frequency of events to the amplitude (left). Quantified averages of event amplitude are displayed for each cell as individual data points and compared between Ctrl (black) and hAPP injected (magenta) conditions (right). L5 SS Ctx mEPSCs no amplitude change (p=0.7660, t=0.3058, df=10). **Bottom:** Cumulative distribution curve for miniature IPSCs in the SS Ctx showing the relationship of relative frequency of events to the amplitude (left). Quantified averages of event amplitude are displayed for each cell as individual data points and compared between Ctrl (black) and hAPP injected (magenta) conditions (right). L5 SS Ctx mIPSCs show an unchanged amplitude (p=0.77766, t=0.2915, df=10). For a, b, c, d, e, f: Individual data points and box plots are displayed. Statistical significance is denoted as *=p<0.05, as determined by two-tailed unpaired t-test

**Extended Data Figure 10.**
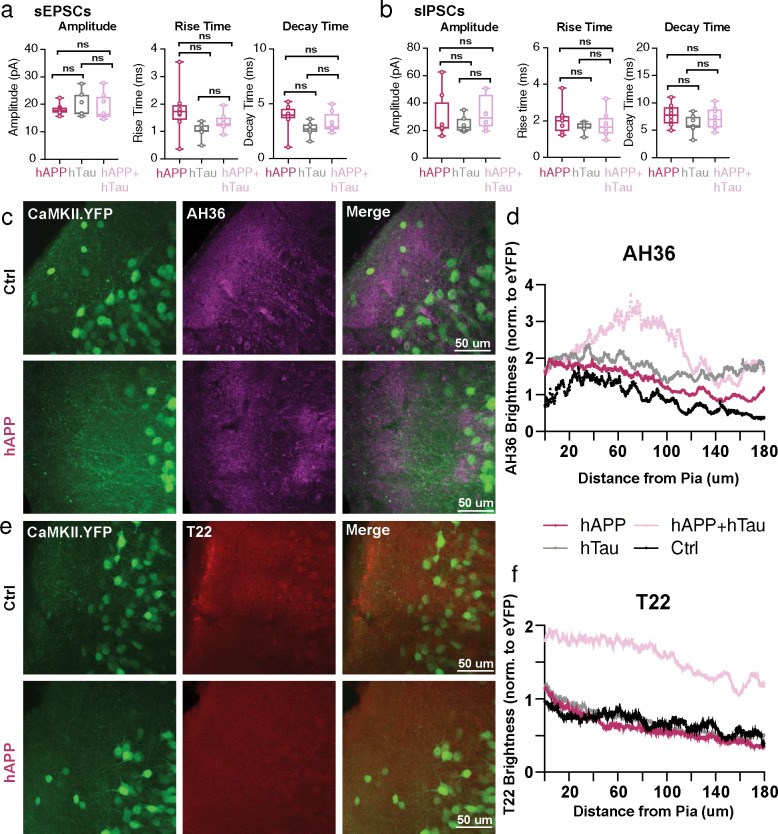
hTau co-injection with hAPP spontaneous properties and Ctrl IHC images **a.** Summary data of sEPSC properties. sEPSC properties between hAPP injection, hTau injection, or hAPP+hTau injection are not significantly different (Amplitude: hAPP vs. hTau p=0.7491, hAPP vs. hAPP+hTau p=0.9387, hTau vs. hAPP +hTau p=0.9153; Rise Time: hAPP vs. hTau p=0.0513, hAPP vs. hAPP+hTau p=0.3070, hTau vs. hAPP +hTau p=0.5506; Decay Time: hAPP vs. hTau p=0.1144, hAPP vs. hAPP+hTau p=0.5379, hTau vs. hAPP +hTau p=0.5478; df=20, One-way ANOVA with Multiple Comparisons). **b.** Summary data of sIPSC properties. sIPSC amplitudes between hAPP injection, hTau injection, or hAPP+hTau injection are not significantly different (Amplitude: hAPP vs. hTau p=0.6886, hAPP vs. hAPP+hTau p=0.8812, hTau vs. hAPP +hTau p=0.4142; Rise Time: hAPP vs. hTau p=0.3924, hAPP vs. hAPP+hTau p=0.6531, hTau vs. hAPP +hTau p=0.8829; Decay Time: hAPP vs. hTau p=0.2064, hAPP vs. hAPP+hTau p=0.7644, hTau vs. hAPP +hTau p=0.5331; df=20, One-way ANOVA with Multiple Comparisons). **c,e.** IHC representative images at 60x magnification for Ctrl (top) or hAPP (bottom) injected mice with staining for either AH36 (c) or T22 (e). **d.** Ctrl, hAPP, hTau, and hAPP+hTau were analyzed for AH36 brightness using four line scans in each slice. AH36 brightness was normalized to CaMKII.eYFP brightness to control for any potential variability in viral expression. hAPP+hTau showed the highest level of AH36 brightness, most notably between 40–120 μm from the pia. **f.** hAPP, hTau, and hAPP+hTau were analyzed for T22 brightness using four line scans in each slice. AH36 brightness was normalized to CaMKII.eYFP brightness to control for any potential variability in viral expression. hAPP+hTau showed a higher level of T22 brightness, above all other groups which displayed only background levels of T22 positivity.

**Extended Data Table 1.** Passive and active properties of LEC and SS Ctx neurons Passive and active properties of principal excitatory cells (top) or tdTom+ PV interneurons (bottom) in L2 LEC and L5 SS Ctx.

Principal cells in LEC and SS Ctx					
Features	Average±SEM LEC EXC	Average±SEM SS Ctx EXC	p-value	t-value	Degrees of Freedom
**AHP (mV)**	9.66±0.75	6.33±0.64	<0.01	3.41	40
**Threshold (mV)**	−41.97±1.18	−44.61 ±1.26	0.14	1.53	40
**dV/dt Max**	353.40±13.97	469.60±31.50	<0.01	3.37	40
**Input Resistance (MΩ)**	103.60±14.51	117.80±8.93	0.40	0.86	40
**Resting Membrane Potential (mV)**	−63.39±1.18	−71.31 ±1.50	<0.01	4.00	40
**Membrane Capacitance (pF)**	205.40±14.68	183.30±12.73	0.26	1.14	40
**Membrane Tau (ms)**	19.65±1.84	20.64±1.74	0.70	0.39	40
**Accomodation Ratio**	2.52±0.47	2.93 ±0.28	0.45	0.77	40
PV Interneurons in LEC and SS Ctx					
Features	Average±SEM LEC PV-IN	Average±SEM SS Ctx PV-IN	p-value	t-value	Degrees of Freedom
**AHP (mV)**	20.68±1.01	19.47±1.19	0.44	0.78	25
**Threshold (mV)**	−40.02±1.14	−46.14±0.55	<0.01	2.47	25
**dV/dt Max**	553.70±29.76	571.60±20.48	0.65	0.46	25
**Input Resistance (MΩ)**	147.30±16.44	121.20±17.1 4	0.29	1.08	25
**Resting Membrane Potential (mV)**	−60.38±1.60	−65.71 ±1.89	0.04	3.15	25
**Membrane Capacitance (pF)**	70.17±5.46	71.91 ±9.51	0.87	0.17	25
**Membrane Tau (ms)**	9.98±0.62	7.39±0.47	<0.01	3.181	25
**Accomodation Ratio**	2.14±0.24	1.71 ±0.09	0.154	1.470	25

All were Unpaired t-tests, Two-tailed

## Data Availability

The PV-interneuron mass spectrometry proteomics data generated will be deposited to the ProteomeXchange Consortium via the PRIDE partner repository. The 2020 mouse Uniprot-database (downloaded from https://www.uniprot.org/help/reference_proteome).
